# Evolutionary and Phylogenetic Analysis of the Hepaciviruses and Pegiviruses

**DOI:** 10.1093/gbe/evv202

**Published:** 2015-10-21

**Authors:** Julien Thézé, Sophia Lowes, Joe Parker, Oliver G. Pybus

**Affiliations:** ^1^ Department of Zoology, University of Oxford, United Kingdom; ^2^ Biodiversity Informatics and Spatial Analysis, The Jodrell Laboratory, Royal Botanic Gardens, Kew, United Kingdom

**Keywords:** hepatitis C virus, human pegivirus, host range, cross-species transmission, recombination, parallel molecular evolution

## Abstract

The known genetic diversity of the hepaciviruses and pegiviruses has increased greatly in recent years through the discovery of viruses related to hepatitis C virus and human pegivirus in bats, bovines, equines, primates, and rodents. Analysis of these new species is important for research into animal models of hepatitis C virus infection and into the zoonotic origins of human viruses. Here, we provide the first systematic phylogenetic and evolutionary analysis of these two genera at the whole-genome level. Phylogenies confirmed that hepatitis C virus is most closely related to viruses from horses whereas human pegiviruses clustered with viruses from African primates. Within each genus, several well-supported lineages were identified and viral diversity was structured by both host species and location of sampling. Recombination analyses provided evidence of interspecific recombination in hepaciviruses, but none in the pegiviruses. Putative mosaic genome structures were identified in NS5B gene region and were supported by multiple tests. The identification of interspecific recombination in the hepaciviruses represents an important evolutionary event that could be clarified by future sampling of novel viruses. We also identified parallel amino acid changes shared by distantly related lineages that infect similar types of host. Notable parallel changes were clustered in the NS3 and NS4B genes and provide a useful starting point for experimental studies of the evolution of *Hepacivirus* host–virus interactions.

## Introduction

Hepaciviruses and pegiviruses are two genera of the viral family *Flaviviridae*. This family comprises a genetically diverse group of viruses, several of which cause significant human diseases, and includes two further genera, the pestiviruses and flaviviruses. Both the *Hepacivirus* and *Pegivirus* genera contain species that infect humans. Hepatitis C is a disease caused by the hepatitis C virus (HCV), a hepacivirus that infects approximately 3% of the world’s population, yet was discovered only comparatively recently, in 1989 (Choo et al. 1989). HCV is one of the most important causes of severe chronic liver disease (Pfaender et al. 2014) and the healthcare costs associated with HCV infection are estimated to be $6.5 billion in the United States alone (Razavi et al. 2013). Human pegivirus (HPgV) is the most closely related human virus to HCV (Stapleton 2003) and is also a recent discovery. HPgV was described only in 1995, at which time it was known as hepatitis G, or GB virus C (Simons, Leary, et al. 1995; Simons, Pilot-Matias, et al. 1995). HPgV is a lymphotropic virus but unlike HCV it has little, if any, associated pathogenicity in humans, although it infects an estimated 5% of people worldwide (Stapleton et al. 2011) and may be of clinical relevance in individuals who are coinfected with human immunodeficiency virus-1 (HIV-1) (Williams et al. 2004).

In recent years there has been a huge expansion in our knowledge of the number, genetic diversity, and host range of *Hepacivirus* and *Pegivirus* species. Since 2010, more than 250 new virus sequences isolated from nonhuman host species have been published. These new viruses have been found in a wide range of mammalian hosts, including bats (Epstein et al. 2010; Quan et al. 2013), primates (Lauck et al. 2013; Sibley et al. 2014), rodents (Drexler et al. 2013; Kapoor, Simmonds, Scheel, et al. 2013; Firth et al. 2014), and domesticated animals such as dogs (Kapoor et al. 2011; El-Attar et al. 2015), cows (Baechlein et al. 2015; Corman et al. 2015), and horses (Burbelo et al. 2012; Lyons et al. 2012; Chandriani et al. 2013; Kapoor, Simmonds, Cullen, et al. 2013; Gemaque et al. 2014; Reuter et al. 2014; Tanaka et al. 2014; Matsuu et al. 2015; Pfaender, Cavalleri, et al. 2015; Scheel et al. 2015). As a result of these discoveries, bats, rodents, and horses are now of significant interest to the hunt for the zoonotic origin of human hepaciviruses and pegiviruses, whereas in the past primates were the primary target of this research (Simmonds 2013). The potential for bat and rodent populations to act as reservoirs of viral infection and sources of cross-species transmission is well known; they have been estimated to be responsible for a quarter of all recently emerged human pathogens (Woolhouse and Gaunt 2007). The recent explosion in the known genetic diversity of the *Hepacivirus* and *Pegivirus* genera suggests that there may be many more viral species in novel host species yet to be discovered, hence the picture of hepacivirus and pegivirus evolution may yet change. Despite this, now is a good time to consolidate the discoveries of the last 3 years.

**Fig. 1.—:**
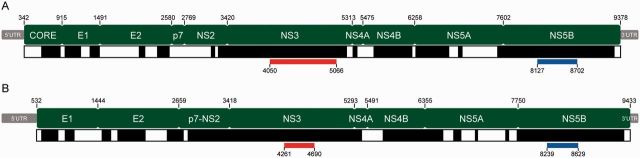
Genome structures (green) of the hepacivirus and pegivirus reference genomes. (*A*) HCV strain H77 and (*B*) simian pegivirus strain NC_001837. Positions of gene boundaries are marked above each structure. Black and white bars below each structure indicate the regions included in the genome-wide alignment (black) and regions that were unalignable (white). The locations of the NS3 and NS5B subgenomic regions that were analyzed separately are indicated in red and blue, respectively.

The genome structures of hepaciviruses and pegiviruses are conserved and share many similarities (fig. 1). Both genera are single-stranded positive sense RNA viruses and their genomes are translated in a single open reading frame as if they were mRNA molecules. The genomes of hepaciviruses and pegiviruses tend to be around 10 kb in length. HCV contains ten distinct genes capped by untranslated regions (UTRs) at the 5′- and 3′-ends: a structural core protein (C), two envelope proteins (E1 and E2), nonstructural assembly proteins (p7 and NS2), and other nonstructural proteins used in replication (NS3, NS4A, NS4B, NS5A, and NS5B) (fig. 1; Moradpour et al. 2007). HPgV has a very similar structure, with several structural and nonstructural proteins. However, not all pegiviruses appear to encode a core protein (Pfaender et al. 2014) and some show evidence of a different, third structural protein, X, between the envelope and nonstructural proteins (fig. 1; Sibley et al. 2014). Despite their conserved genome structure, there is very significant genetic diversity within and among the two genera. This is in part due to highly error-prone replication by the virally encoded RNA polymerase (Neumann et al. 1998).

Hepaciviruses and pegiviruses infect a wide range of mammals, although each virus species tends to have narrow a host range (Sibley et al. 2014). Nonhuman hepaciviruses are of significant interest for at least two reasons. First, very little is known about the zoonotic origin or potential animal reservoirs of HCV. Molecular clock analyses indicate that HCV has infected human populations for hundreds to thousands of years (e.g., Smith et al. 1997; Markov et al. 2012; Iles et al. 2014) even though the disease it causes has been discovered only recently, and a long-term evolutionary association between HCV and humans has also been hypothesized (Simmonds 2013). The origin of HPgV is also unknown, and there is no evidence indicating the animal populations responsible for the transfer of these viruses to humans (Makuwa et al. 2006). Second, there is no animal model in which HCV infection can be studied straightforwardly, and current cell culture systems are limited in scope, so a better understanding of related viruses could provide valuable insights into the biology of HCV (Bukh 2012). Chimpanzees that are experimentally infected with HCV suffer from chronic infection resembling that observed in humans; however, surveys have not found viruses related to HCV in wild chimpanzees (Makuwa et al. 2003). Although chimpanzees are the most realistic model system of human infection available, their use in medical research raises very substantial ethical, logistical, and financial problems (Bukh 2004). More recently, human liver chimeric mice have been used as small animal models of HCV infection (Bissig et al. 2010) but their lack of functional or natural immune system limits their use (Bukh 2012). In the future nonhuman hepaciviruses, notably equine hepaciviruses, may prove to be practical as animal models for HCV biology (Pfaender, Cavalleri, et al. 2015).

Despite numerous recent reports of newly discovered species of hepaciviruses and pegiviruses (see above), a systematic phylogenetic and molecular evolutionary analysis of the two genera has not been conducted. This has resulted incongruent tree topologies being reported for the *Hepacivirus* genus among recent studies (Kapoor, Simmonds, Scheel, et al. 2013; Quan et al. 2013; Tanaka et al. 2014; Baechlein et al. 2015; Corman et al. 2015). Previous phylogenetic analysis have utilized only small regions of the virus genome (specifically, parts of the NS3 and NS5B genes), or have investigated only a subset of available taxa. Further, no analysis of recombination or parallel molecular evolution at the interspecific level has been undertaken. Although recombination has been reported between genotypes and subtypes of HCV, it does not appear to be a significant process in HCV evolution (Kalinina et al. 2002; Colina 2004; Cristina and Colina 2006; Raghwani et al. 2012; Shi et al. 2012; Galli and Bukh 2014), although it has been hypothesized that ancient recombination may explain the origin of pegivirus-like internal ribosomal entry sites in rodent hepaciviruses (located in the 5′-UTR of *Flaviviridae*) (Drexler et al. 2013). In this study, we perform a comprehensive evolutionary analysis of the hepaciviruses and pegiviruses that draws together all currently available data. We attempt to resolve the phylogenetic structure of both genera and we find that taxa appear to cluster most strongly by host species type. Intriguingly, we find some evidence for interspecific recombination in the hepaciviruses, although clear interpretation of this result is hampered by viral genetic diversity and undersampling. Further, we detect a number of important parallel amino acid mutations among hepacivirus lineages that infect similar hosts, which suggests potential adaptive residues suitable for investigation in experimental studies.

## Materials and Methods

### Sequence Data

All currently available hepacivirus and pegivirus sequences were collated from GenBank and EMBL public databases. These searches returned many thousands of sequences but the vast majority represented HCV, and to a lesser extent, HPgV, so these two viruses were excluded from the initial search results. Instead, one representative genome from each genotype of HCV and HPgV was selected for inclusion in the data set. Biological information obtained for each sequence included accession number, host species, isolate name, and country of collection (supplementary tables S1 and Supplementary Data, Supplementary Material online).

### Multiple Alignment and Phylogenetic Inference

Separate amino acid alignments were generated for hepacivirus and pegivirus complete coding sequences. Alignments were constructed using the Mafft program (Katoh and Standley 2013) followed by substantial manual editing using AliView (Larsson 2014). We then used the BMGE program (Criscuolo and Gribaldo 2010) to trim the multiple amino acids alignments prior to phylogenetic analysis, in order to remove poorly aligned genomic regions (see supplementary materials S1 and Supplementary Data, Supplementary Material online). After trimming the hepacivirus and pegivirus alignments were 1,927 and 2,233 amino acids in length, which represents 64% and 74% of their total coding regions, respectively (fig. 1). These alignments are hereafter termed the “genome-wide” alignments. Maximum likelihood (ML) phylogenies were estimated from the genome-wide amino acids alignments using the LG + I + G + F substitution model and parameters; this model was selected under the Aikaike information criterion using the ProtTest program (Darriba et al. 2011). ML phylogenies were estimated using RAxML (Stamatakis 2006). Statistical support for phylogenetic nodes was assessed using a bootstrap approach (with 100 replicates). Midpoint rooting was chosen to root ML trees in order to avoid long-branch attraction with highly divergent outgroups.

Many of the sequences obtained represented small subgenomic regions, not whole genomes. Two genomic regions in particular were commonly sequenced in both genera: part of NS3, a viral helicase, and of NS5B, the viral RNA-dependent RNA polymerase. These genes correspond to strongly conserved regions within the genome-wide alignment (fig. 1). Multiple alignments of partial NS3 and NS5B proteins contain substantially less phylogenetic information but include a wider range of taxa. Since there is a trade-off between the number of taxa and sequence length, we chose to examine both genome-wide and subgenomic data sets. We therefore performed multiple amino acids alignment and ML phylogenetic inference, as above, on both the partial NS3 and NS5B regions, using the LG + I + G substitution model and parameters, as selected by the ProtTest program.

### Recombination Analyses

To investigate the possibility of interspecific virus recombination, we used a combination of recombination analysis methods and partitioned phylogenies. Prior to recombination analysis, we converted the genome-wide amino acids alignments to codon-based alignments. Saturation tests were performed in Dambe (Xia 2013), which implements the Xia et al.’s test of nucleotide substitution saturation (Xia et al. 2003; Xia and Lemey 2009). This indicated that all codon-based alignments were saturated at the third codon position. However, once third codon positions were removed, first and second codon positions showed little saturation so these positions were retained and used in the following analyses.

**Fig. 2.—:**
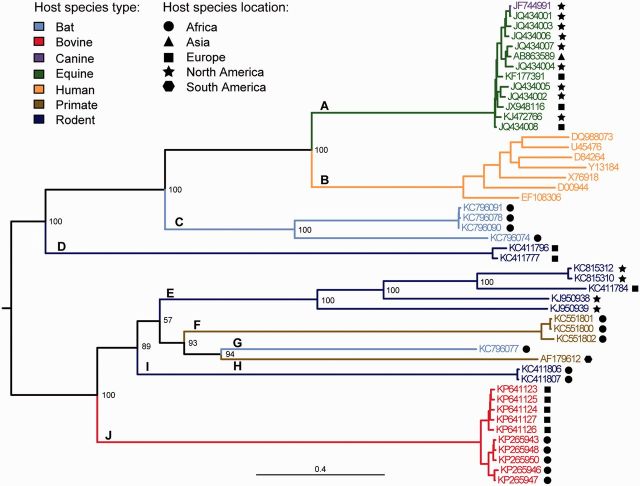
Phylogeny of the Hepaciviruses. Estimated from the genome-wide amino acid alignment using ML inference. Statistical support for phylogenetic nodes was assessed using a bootstrap approach (100 replicates). Tip and branch labels are colored by host species type while the sampling location of nonhuman hosts is denoted by the adjacent symbol. Letters indicate the different *Hepacivirus* lineages discussed in the main text.

**Fig. 3.—:**
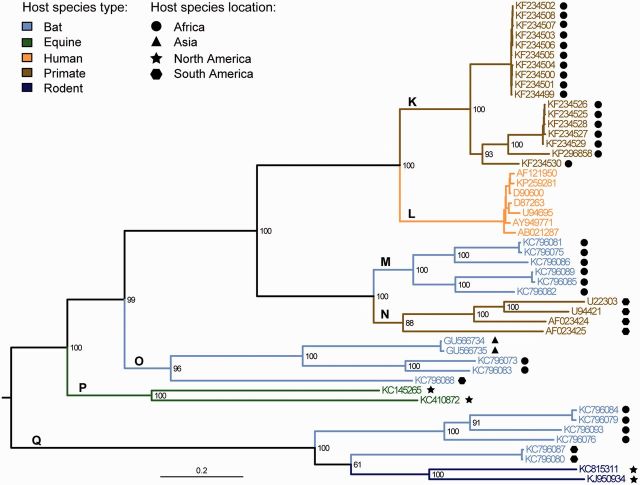
Phylogeny of the Pegiviruses. Estimated from the genome-wide amino acid alignment using ML inference. Statistical support for phylogenetic nodes was assessed using a bootstrap approach (100 replicates). Tip and branch labels are colored by host species type while the sampling location of nonhuman hosts is denoted by the adjacent symbol. Letters indicate the different *Pegivirus* lineages discussed in the main text.

To facilitate the analysis of interspecific recombination, taxa in the original data sets were subsampled in a phylogenetically informed manner. Specifically, a single representative was randomly chosen from each defined lineage in the ML phylogenies of hepaciviruses (lineages A–J in fig. 2) and pegiviruses (lineages K–Q in fig. 3). For the hepaciviruses, these were sequences U45476, KC796090, JQ434007, KC411806, KC796077, KC411777, KC815312, KC551802, AF179612, and KP265943, and for the pegiviruses the representatives were U94695, KC796075, KC796087, KC410872, KC796088, KF234499, and U22303.

We first analyzed the data using RECCO (Maydt and Lengauer 2006), which provides an initial suggestion of whether recombination might have occurred and approximates the location of breakpoints using cost optimization. Only those putative recombinant sequences that produced a cost saving greater than 20 (the mutation cost saved by each additional unit of recombination cost; (Maydt and Lengauer 2006) and which had a *P* value <0.001 were deemed, conservatively, to be worth investigating further.

Sequences that met these conditions underwent additional investigation using the RDP4 program (Martin et al. 2015), which implements multiple methods of recombination detection including GENECOV (Padidam et al. 1999), Bootscan (Martin et al. 2005), 3SEQ (Boni et al. 2007), Chimaera (Posada and Crandall 2001), and MaxChi (Maynard-Smith 1992). We primarily considered the results of the Bootscan analysis, which identifies well supported phylogenetic incongruencies among different portions of the genome (Boni et al. 2010). However, Bootscan and each of the other methods carry their own strengths and weaknesses, so positive results across multiple tests provide the best support for recombination.

In order to examine potential recombination events even more closely, multiple phylogenies were estimated from genomic regions either side of the putative recombination breakpoints. For each potential recombinant lineage, we estimated a ML phylogenetic tree from the proposed recombinant region within the genome-wide multiple alignment, while fixing the topology of the genome-wide phylogeny to that previously estimated, with the exception of the lineage under investigation, whose location was free to vary. Potential recombinant lineages will change their position in the phylogeny if recombination has taken place.

### Parallel Mutation Analysis

We sought to identify parallel amino acids changes along ancestral lineages in the hepacivirus and pegivirus phylogenies. We focused on internal branches that were basal to groups of virus species that infect the same type of host (e.g., humans, primates, rodents, and bats). In this analysis, we grouped horses and bovines as a single category of host, as only one virus lineage from each was available for analysis. Although equids and bovids are not closely related they do share a similar ecological relationship with humans through domestication that may play a role in cross-species transmission. A computer program (available on available on GitHub with basic documentation at https://github.com/lonelyjoeparker/qmul-genome-convergence-pipeline) was used to identify parallel amino acid changes, that is, those that are present in all viruses descended from the ancestral branches defined above. Amino acid changes were ignored if they were seen in other sequences, that is, those that were not descendents of the ancestral lineages in question.

## Results

### Phylogenetic Analysis of Hepaciviruses

ML phylogenies of the hepaciviruses (fig. 2 and supplementary figs. S1 and Supplementary Data, Supplementary Material online) indicated the presence of ten distinct lineages (A–J), almost all of which are supported by high bootstrap scores in the phylogeny of the genome-wide alignment (fig. 2) but by lower bootstrap scores in trees estimated from the more conserved and shorter NS3 and NS5B alignments (supplementary figs. S1 and Supplementary Data, Supplementary Material online). Lineage A contains equine and canine hepaciviruses. Lineage B contains the HCV (from humans) while lineage C comprises viruses isolated from Kenyan bats. Lineages D, E, and I all contain viruses from rodent host species. Lineage F contains hepaciviruses from colobus monkeys and lineage G contains a single virus found in African bats. Lineage H corresponds to the GB virus B which was isolated from captive tamarins (Simons, Leary, et al. 1995; Simons, Pilot-Matias, et al. 1995). Finally, lineage J represents the recently discovered hepacivirus lineage in cattle.

The phylogeny of the genome-wide hepacivirus alignment (fig. 2) shows two distinct monophyletic clades (upper and lower; defined by the most basal well-supported split), comprising lineages A–D and E–J, respectively. Only viruses from bats and rodents are found in both the upper and lower clades. As expected, HCV is a sister lineage to the equine and canine hepaciviruses. The new cattle hepacivirus lineage (J) is an outgroup of the lower clade. Taxa within most lineages are genetically relatively similar, with the exception of the bat viruses in lineage C, the rodent viruses in lineage E, and HCV (lineage B). The lowest bootstrap scores are observed for lineages E and I, which are separated by a short internal branch (fig. 2).

Comparing the genome-wide hepacivirus phylogeny (fig. 2) to that estimated from partial NS3 sequences (supplementary fig. S1, Supplementary Material online), we can see that the topology of the upper clade is maintained. However there are differences in the topology of the lower clade, for example, lineages J and F are now sister lineages, and the outgroup of the lower clade is lineage I (supplementary fig. S1, Supplementary Material online). However, bootstrap support for these arrangements are weak (<70%; supplementary fig. S1, Supplementary Material online). The partial NS5B phylogeny (supplementary fig. S2, Supplementary Material online) shows further topological differences from the genome-wide tree. Most notably, lineage G now appears as an outgroup to lineages A and B (bootstrap score = 94%) whereas C is most closely related to lineage F (bootstrap score = 86%). Other topology changes are present in the partial NS5B tree but none are supported by bootstrap scores >80% (supplementary fig. S1, Supplementary Material online). Some differences between the genome-wide and subgenomic phylogenies may be due to random error or long-branch attraction. However, the well supported rearrangements seen in the partial NS5B tree suggest that detailed analysis of potential recombination in the hepaciviruses is warranted.

We also examined the host species location of viruses in the hepacivirus genome-wide phylogeny; taxa were labeled by continent of the host species from which they were isolated (fig. 2 and supplementary figs. S1 and Supplementary Data, Supplementary Material online). HCV was ignored because its global distribution is highly complex and the result of recent anthropogenic factors (Messina et al. 2015). The same may also be true for the equine and canine hepaciviruses, as their sequences exhibit a scattered spatial distribution despite showing little genetic divergence. Domesticated horses, especially racehorses, are transported worldwide, and this may have disguised any previous geographic pattern that arose from natural processes. The other hepaciviruses lineages are, in general, isolated from hosts from the same continent, except for lineages E, I, and J. Lineages E and I contain diverse viruses found in African, European, and North American rodents whereas lineage J contains viruses isolated in African and European cattle.

### Phylogenetic Analysis of Pegiviruses

Seven lineages (K–Q) were evident in the ML phylogenies of the pegiviruses (fig. 3 and supplementary figs. S3 and Supplementary Data, Supplementary Material online). Simian pegiviruses are found in lineage K and HPgV (formerly GB-virus C) comprises lineage L. Lineage M contains viruses from African and Asian bats of various species, whereas lineage O contains bat viruses from multiple continents. Lineage N contains sequences of GB virus A from South American primates (Simons, Leary, et al. 1995; Simons, Pilot-Matias, et al. 1995), now known as simian pegiviruses (Stapleton et al. 2011). Lineage P represents equine pegivirus species from Europe and North America and lineage Q contains rodent and bat virus viruses sampled in Africa and North and South America.

The genome-wide pegivirus phylogeny shows that HPgV is a well-supported sister group of the African simian pegiviruses (lineages K and L; fig. 3). Lineages M and N are also closely related. As in the hepacivirus genome-wide tree (fig. 2), most lineages are separated by long internal branches and lineages are supported by high bootstrap values.

If we compare the genome-wide pegivirus phylogeny (fig. 3) to the trees estimated from the partial NS3 and NS5B regions (supplementary figs. S3 and Supplementary Data, Supplementary Material online), then the clustering of lineages K and L with a third lineage, MN, is conserved. However, in the NS3 and NS5B phylogenies, viruses that were isolated from chimpanzees (and which are not present in the genome-wide alignment) are situated basal to the HPgVs (supplementary figs. S3 and Supplementary Data, Supplementary Material online), indicating that the latter are derived from the paraphyletic simian pegiviruses. Further, in the NS3 and NS5B phylogenies, lineages P and O are sister groups (supplementary figs. S3 and Supplementary Data, Supplementary Material online) rather than being paraphyletic with respect to each other, as they are in the genome-wide tree (fig. 3). Among-lineage bootstrap values are higher in the NS3 and NS5B pegivirus phylogenies (supplementary figs. S3 and Supplementary Data, Supplementary Material online) than in the corresponding hepacivirus trees (supplementary figs. S1 and Supplementary Data, Supplementary Material online) and the pegiviruses exhibit fewer topological changes among the different phylogenies.

Many of the non-HPgV s were sampled in Africa and only the equine pegiviruses were isolated in Europe. As for the hepaciviruses, more closely related sequences tend to share the same continent of sampling, but there is little discernable pattern at the among-lineage level.

### Recombination Analyses

No significant recombination breakpoints were detected in the pegiviruses using the program RECCO, so further recombination analysis of that genus was not pursued. However, significant results were obtained for the representatives of three hepacivirus lineages (C, F, and G). Two breakpoints were detected very close together (at positions 6570 and 6576; all positions are relative to the H77 HCV reference strain; fig. 1) in an African colobus monkey virus (accession number KC551802) belonging to lineage F, so these likely represent one breakpoint. Other breakpoints were detected at nearby positions 6762 in sequence KC796090 (bat virus, lineage C) and 6906 in sequence KC796077 (bat virus, lineage G). Thus RECCO identified multiple potential recombination breakpoints in a region of the alignment that corresponds to the middle of the NS5A protein (positions 6570–6906; fig. 1).

The proposed recombinants identified by RECCO were subsequently analyzed in detail using the methods implemented in RDP (table 1). In the analysis of lineages C, F, and G, one recombination event was detected using Bootscan analysis for each lineage, with a high bootstrap support (>80%). Each putative recombination event had an associated binomial *P* value < 0.00001, representing the probability that, in the specified region, the recombinant is more closely related to the minor parent than the major parent by chance alone (the minor parent is the apparent contributor of smaller recombinant fragment, while the major parent is the apparent contributor of the rest of the sequence). Moreover RDP also returned positive results for each lineage using a number of other methods (RDP, GENECOV, MaxChi, Chimaera, and SiScan), providing further support for these putative recombination events.

**Table 1:** RDP Results from the Hepacivirus Lineage Representatives

Recombinant Lineage	Sequence	Binomial *P* Value	Bootstrap Support (%)	Major Parent Lineage	Minor Parent Lineage	Positive in Programs	Recombinant Positions (H77 Reference)
C	KC796090	2.9 × 10^−9^	87	A	F	RDP Bootscan MaxChi Chimaera SiScan	8091–8618
F	KC551802	1.2 × 10^−7^	83	G	J	RDP GENECONV Bootscan MaxChi Chimaera SiScan	8262–8834
G	KC796077	4.9 × 10^−6^	86	H	A	RDP Bootscan MaxChi Chimaera SiScan	8205–8480

Bootscan estimated that the 5′ recombination breakpoints were located between positions 8091 and 8262 and the 3′-breakpoints were sited between positions 8480 and 8834 (positions relative to reference strain H77; fig. 1). The 99% confidence limits of these positions were not determined, meaning that RDP had difficulties in identifying the breakpoint positions. These estimated 5′ breakpoints positions are approximately 1,000–1,500 nt downstream of those detected with RECCO. However, the two sets of locations are sited either side of a large unalignable region in the genome-wide alignment (fig. 1); the absence of this region from the alignment means that small amounts of random estimation error could lead to substantial jumps in estimated breakpoint placement. Further, all 3′ breakpoint locations were in the same region of the hepacivirus genome, corresponding to the middle of NS5B. Because RDP implements a more sophisticated suite of tools for estimating breakpoint locations than RECCO, we rely on the results of the former and conclude that the recombinant fragments most likely lie between the 5′ boundary and the middle of NS5B.

The hypothesized major parent lineages for each putative recombinant are consistent with the hepacivirus phylogeny estimated from the genome-wide alignment (fig. 2). The putative minor parent of KC551802 is lineage J (its major parent is lineage F); lineages J and F are both located in the lower clade of the hepacivirus tree (fig. 2 and table 1). In contrast, the putative minor parent lineage of KC796077 is lineage A, which is more distant from its major parent (lineage G) in the hepacivirus phylogeny (fig. 2 and table 1). A similar discrepancy is seen for KC796090, whose putative minor parent is lineage F and whose major parent is lineage C (table 1). The consensus scores for these events are relatively high (>0.45), suggesting that RDP has determined the recombinant and putative parental sequences reasonably reliably.

**Fig. 4.—:**
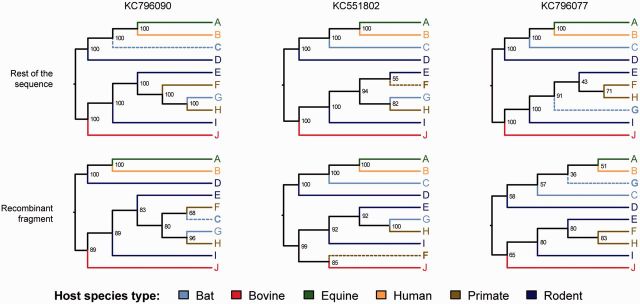
Tree topologies of recombinant hepacivirus lineages. Trees were estimated from the putative recombinant fragment (bottom row) and from remainder of the genome (top row) using ML. In each case the tree topology was fixed for all sequences except the putative recombinant sequence in question (shown along top, and highlighted in bold and with dashed branch line in each tree). Tip and branch labels are colored by host species type.

These results were further explored using phylogenetic analysis. For each of the three putative recombinant lineages, two phylogenetic trees were estimated, one from the proposed recombinant fragment, and one from the remainder of the genome-wide alignment (fig. 4). The putative recombinant region of KC551802 is closely related to lineage J, consistent with the RDP results. Given the long branches leading to two lineages J and F, this observation could result from random error or long-branch attraction rather than recombination. However, for KC796077 and KC796090, the topology of trees estimated for the two genome regions are substantially different and, in both cases, the putative recombinant fragment jumps between the upper and lower clades of the hepacivirus phylogeny. This is again consistent with the RDP results. Potential recombination between lineages C and G is particularly interesting because both lineages were isolated from bat species from Kenya.

### Parallel Mutation Analysis

We analyzed the genome-wide hepacivirus and pegivirus alignments (supplementary materials S1 and Supplementary Data, Supplementary Material online) to identify amino acid mutations that occurred on multiple branches basal to the defined lineages (table 2). Strikingly, we identified 50 such parallel amino acid changes among lineages in the *Hepacivirus* genus, whereas only three were found for the *Pegivirus* genus. One theoretical explanation for this difference is a higher rate of recombination in hepaciviruses, which could generate a greater number of apparent homoplasies (Maynard Smith and Smith 1998). Of the parallel mutations found in the hepaciviruses, a disproportionate number are found in the NS4B protein (ten mutations in a region only ∼250 amino acids long), which acts as membrane anchor for the replication complex (Chevaliez and Pawlotsky 2006). Moreover, the only parallel amino acid change that was observed in multiple pairwise comparisons (position 6210 in hepacivirus reference genome H77; fig. 1) is located in the NS4B protein. The hepacivirus NS3 and NS5B regions also exhibit a high density of parallel mutations (15 and 13, respectively).

**Table 2:** Parallel Amino Acid Changes among Hepacivirus and Pegivirus Lineages

Genus	Host Species Type Comparison	Lineages	Region	Position (H77/NC_001837 References)	Amino Acid (Derived Change)
*Hepacivirus*	Equine–Bovine	A–J	E1	977	L
			NS4B	5748	R
			NS4B	5760	A
			NS4B	6210	V
			NS4B	6216	T
			NS5B	8364	N
			NS5B	8571	T
	
	Human–Primate	B–F	CORE	789	A
			NS3	4776	Q
			NS3	5106	Y
		
		B–H	NS3	4680	D
			NS5A	6363	F
			NS5A	6429	P
			NS5A	7557	S
	
		B–F–H	NS3	4923	Y
	
	Bat–Bat	C–G	NS3	3855	A
			NS5A	6468	V
			NS5B	7722	D
			NS5B	8334	M
	
	Rodent–Rodent	D–E	E1	1434	N
			NS4B	5916	T
			NS4B	6210	F
		
		D–I	E2	2367	A
			E2	2520	V
			NS2	3183	W
			NS2	3204	D
			NS2	3330	N
			NS3	3480	I
			NS3	3825	R
			NS3	3888	V
			NS3	3975	Q
			NS3	3981	A
			NS3	4773	Q
			NS3	4794	I
			NS4B	5733	V
			NS4B	5991	A
			NS4B	6063	D
			NS5B	7836	R
			NS5B	7854	F
			NS5B	8091	P
			NS5B	8376	T
			NS5B	8427	V
			NS5B	8943	W
			NS5B	8973	H
		
		E–I	E1	1071	M
			NS3	3882	L
			NS4B	6120	T
			NS5A	6327	R
			NS5B	8814	L
		
		D–E–I	NS3	3726	E
*Pegivirus*	Primate–Primate	K–N	NA	NA	NA
	
	Human–Primate	L–N	E2	2512	E
			NS5B	8230	I
		K–L–N	NS3	4174	T
	
	Bat–Bat	M–O	NA	NA	NA
		M–Q	NA	NA	NA
		O–Q	NA	NA	NA
		M–O–Q	NA	NA	NA

Parallel mutations are of particular interest if they occur on genetically distinct lineages that infect related hosts, as these are less likely to arise by chance and may represent viral adaptations to specific types of hosts. In the hepaciviruses, we identified one parallel amino acid mutation shared by three branches basal to human/primate viruses (lineages B, F, and H; position 4923 in hepacivirus reference genome H77; fig. 1) and one change shared by three branches basal to rodent viruses (lineages D, E, and I; position 3726 in hepacivirus reference genome H77; fig. 1). For the pegiviruses we found one mutation along that was parallel along three branches basal to human/primate viruses (lineages K, L and N; position 4174 in pegivirus reference genome NC_001837). All of these multiply parallel changes were in the NS3 protein.

## Discussion

It has long been recognized that an understanding of the evolution and zoonotic origins of viruses can have important consequences for public health and improve our understanding of infection and pathogenesis, as demonstrated by research on simian immunodeficiency viruses (SIV), poxviruses, and herpesviruses (Zak and Sande 1999). Although a slew of recent papers have reported new hepaciviruses and pegiviruses, ours is first known study to collate and synthesize these findings and to systematically analyze the complete known diversity of these genera at the genome-wide level.

Bats have been suggested to be a reservoir of both hepaciviruses and pegiviruses due to their basal position in phylogenetic trees and the paraphyletic grouping of bat pegiviruses (Quan et al. 2013). The genetic distances between HCV and HPgV and the bat viruses most closely related to them are large, so the currently known hepaciviruses and pegiviruses from bats are unlikely to represent the virus populations directly responsible for zoonotic transmission. Some zoonotic viruses have been found to require a “stepping stone” species to facilitate transfer between bats and humans. For example, horseshoe bats were discovered to be the source of severe acute respiratory syndrome, whereas civet cats, which had previously been assumed to be basal, were relegated to the position of an intermediate host species (Lau et al. 2005). Importantly, the phylogenetic distribution of bat hepaciviruses and pegiviruses could yet change with more sampling; the long internal branches in the phylogenies of these groups (figs. 2 and 3) may represent massive undersampling of true virus diversity (Pybus and Gray 2013).

The same argument can be made for rodents, which appear to host a wide variety of genetically diverse hepaciviruses, found in three separate lineages (fig. 2). Rodent pegiviruses are currently more limited in number, with only two complete genomes available (fig. 3). Although analysis of partial NS3 and NS5B sequences provided more sequences, further sampling of rodent hepacivirus and pegiviruses would greatly enhance our understanding of their evolutionary history and host distribution. The recent discovery of hepaciviruses and pegiviruses in commensal rat species is particularly intriguing, as they represent a population with considerable direct and indirect interactions with humans (Firth et al. 2014).

We might hypothesize that the close relationship between HCV and equine hepaciviruses reflects an ecological link between humans and horses: domesticated horses were, up until the twentieth century, a primary means of transport in many locations. This might have increased the chance of cross-species transmission between these two groups, but the direction of any hypothetical transfer is unknown (Pfaender, Walter, et al. 2015). In addition, there could be intermediate hosts between humans and horses, as the branches separating HCV and equine hepaciviruses are not short. Only further sampling will be able to resolve this.

No close relationship between human and equine viruses is seen in the Pegiviruses. Instead, the pegiviruses exhibit a pattern more similar to that observed for HIV and SIV, with viruses most closely related to HPgV being identified in African primates. SIV is thought to have been transmitted to humans through the hunting or butchering of bushmeat. The primate species from which pegivirus complete genomes are available (fig. 3), particularly the Ugandan red colobus monkey, *Piliocolobus tephrosceles*, are not commonly hunted for bush meat in Uganda (Chapman and Lambert 2000). However the NS3 and NS5B phylogenies show virus isolates from chimpanzees situated basal to the HPgV (supplementary figs. S3 and Supplementary Data, Supplementary Material online), supporting the hypothesis that HPgV originated in chimpanzees or cospeciated within the great apes. This highlights the need to undertake complete genome sequencing of the chimpanzee isolates for which only NS3 or NS5B sequences are available to better understand the evolution of these viruses.

Despite the discovery of many new hepaciviruses and pegiviruses in recent years, there are significant issues regarding sampling diversity. Undersampling of viral diversity is the most likely cause of the long internal branches that generate distinct lineages, each of which contain clusters of closely related viruses. There is surely a huge diversity of hepaciviruses and pegiviruses yet to be discovered, and the species examined to date represent only a small proportion of potential hosts. For example, rodents represent 40% of extant mammalian species (Gorbunova et al. 2014) and bats contribute another 20% of species (Rose and Archibald 2005). However, so far, only 22 species of bat have had hepaciviruses or pegiviruses isolated from them, representing less than 2% of the total number of bat species (Quan et al. 2013). Sampling is even sparser for rodents; hepaciviruses or pegiviruses have been collected from only six rodent species, accounting for approximately 0.25% of the total number of rodent species (Drexler et al. 2013; Firth et al. 2014). The six rodent host species identified so far come from only two of the five suborders of rodent: five are from the Myomorpha suborder and one from the Castorimorpha suborder. Yet even within the narrow range of species sampled, the diversity of viruses discovered in bats and rodents far outweighs that found in humans and horses (Pybus and Gray 2013). If hepaciviruses and pegiviruses are species-specific (Kapoor, Simmonds, Scheel, et al. 2013) then we would expect to find many more virus species once a wider range of bat and rodent species are sampled. Inclusion of more virus species will likely break down the long internal branches in the phylogenies (figs. 2 and 3), providing a more accurate picture of the evolution of these genera. A high level of undersampling is by no means unique to these two genera; it has been estimated that within just nine viral families there is likely to be a vast diversity of viral species yet to be discovered in mammals (Anthony et al. 2013). If new viruses are found that fall basal to the currently known hepaciviruses and pegiviruses, then we would expect that the original criteria for defining these two sister genera (Stapleton et al. 2011) may be questioned and re-evaluated in the future.

Although several tests for interspecific recombination within the hepaciviruses produced significant results (e.g., RECCO, Bootscan), it is hard to produce high bootstrap scores for phylogenetic analyses of recombination when the sequences in question are highly divergent. In influenza viruses it has been suggested that a combination of among-gene and among-lineage evolutionary rate variation can give a false appearance of recombination (Worobey et al. 2002). However, evidence for recombination is strengthened when positive results are produced by multiple analyses. The hypothesized recombination in NS5B gene region between lineages C and G is especially intriguing as these lineages infect the same type of host (Bats) and are spatially overlapping (Kenya). However, we cannot exclude the possibility that the putative recombination events identified in silico in this study are the result of laboratory-generated recombination. As in all such cases, computational analysis alone cannot resolve this issue. Confirmation of recombination could be achieved by resequencing of the proposed recombinant breakpoints from fresh extractions of the initial samples. Further sampling of diverse hepaciviruses will help to answer questions about the rate and nature of recombination in the group with greater confidence.

Our analysis of parallel mutation may also support the notion that hepaciviruses are subject to recombination, as more homoplasies were observed between distantly related hepacivirus lineages than among pegivirus lineages. The higher density of parallel changes found in the hepacivirus NS3, NS4B, and NS5B genes indicate that these genes may play an important role in host-species viral adaptation. The most notable result is our identification of multiply parallel amino acid changes in the NS3 gene leading to lineages infecting the same type of host but not in other lineages. This result is analogous to one previously reported for HIV (Wain et al. 2007), where a parallel change in the viral gag protein was found in three independent lineages leading to HIV groups M, N, and O in humans, but not in the viral ancestors of these groups (SIV isolated from chimpanzees and gorillas). Moreover, the NS3 protein is particularly interesting in terms of host–virus coevolution, as the NS3-4A protease is capable of cleaving human mitochondrial antiviral-signaling protein (MAVS) (Patel et al. 2012), which is necessary for the activation of transcription factors that regulate expression of beta interferon and contributes to antiviral immunity. Two recent studies have shown that the equine hepacivirus NS3-4A protease can cleave human MAVS (Parera et al. 2012; Scheel et al. 2015), questioning the strength of coevolution between these host and viral proteins. However, it remains unclear whether either equine hepacivirus or HCV proteases are capable of cleaving equine MAVS. Hence, the genetic similarity of HCV and equine hepaciviruses may explain why equine hepacivirus NS3-4 protease is capable of cleaving human MAVS.

Previous studies have analyzed the phylogenetic history of the hepaciviruses and pegiviruses using partial NS3 and NS5B gene sequences (Drexler et al. 2013; Quan et al. 2013). These genes are highly conserved due to their importance in viral replication, making them easier and quicker to identify in new host species using PCR and the number of sequences for these regions is correspondingly greater than for whole genomes. Here, in order to maximize phylogenetic information, we estimated phylogenies from all alignable regions with the hepacivirus and pegivirus genomes. These regions comprised 64% of the hepacivirus genome and 74% of the pegivirus genome (fig. 1). As expected, the commonly sequenced partial NS3 and NS5B regions could be easily aligned, but much less of the envelope genes (E1 and E2) and NS5A could be reliably aligned. Further sampling of hepaciviruses and pegivirus diversity may unlock the evolutionary information concealed in regions that are currently unalignable.

While there have been significant advances in the identification and classification of hepaciviruses and pegiviruses, there is clearly still much to be discovered regarding these genera. Further sampling is needed to improve the reliability of sequence alignments and phylogenetic analyses. It would be interesting also to examine the distribution of evidence for positive selection across viral genomes, using dN/dS methods for example, and to test whether conserved regions and diverse genome regions occur in the same location across all viral lineages. Furthermore, both hepaciviruses and pegiviruses tend to be species-specific, indicating that they are well adapted to their hosts (Sawyer and Elde 2012). The sites exhibiting parallel evolution identified here will provide a useful starting point for experimental studies of species-specific replication and the evolution of host–virus interactions. Further investigation in future research of the amino acid changes, we have identified will shed light on the mechanisms of cross-species transmission and may prove useful to those developing a practical animal model of hepacivirus and pegivirus infection.

## Supplementary Material

Supplementary Data
