# Supplementary material for: Evolutionary and Phylogenetic Analysis of the Hepaciviruses and Pegiviruses
Source: Genome Biol Evol. 2015 Oct 21;7(11):2996–3008. doi: 10.1093/gbe/evv202 (PMC5635594; doi:10.1093/gbe/evv202)
Supplement: Supplementary Data [file evv202_Supplementary_Data.zip › FigureS4.pdf]

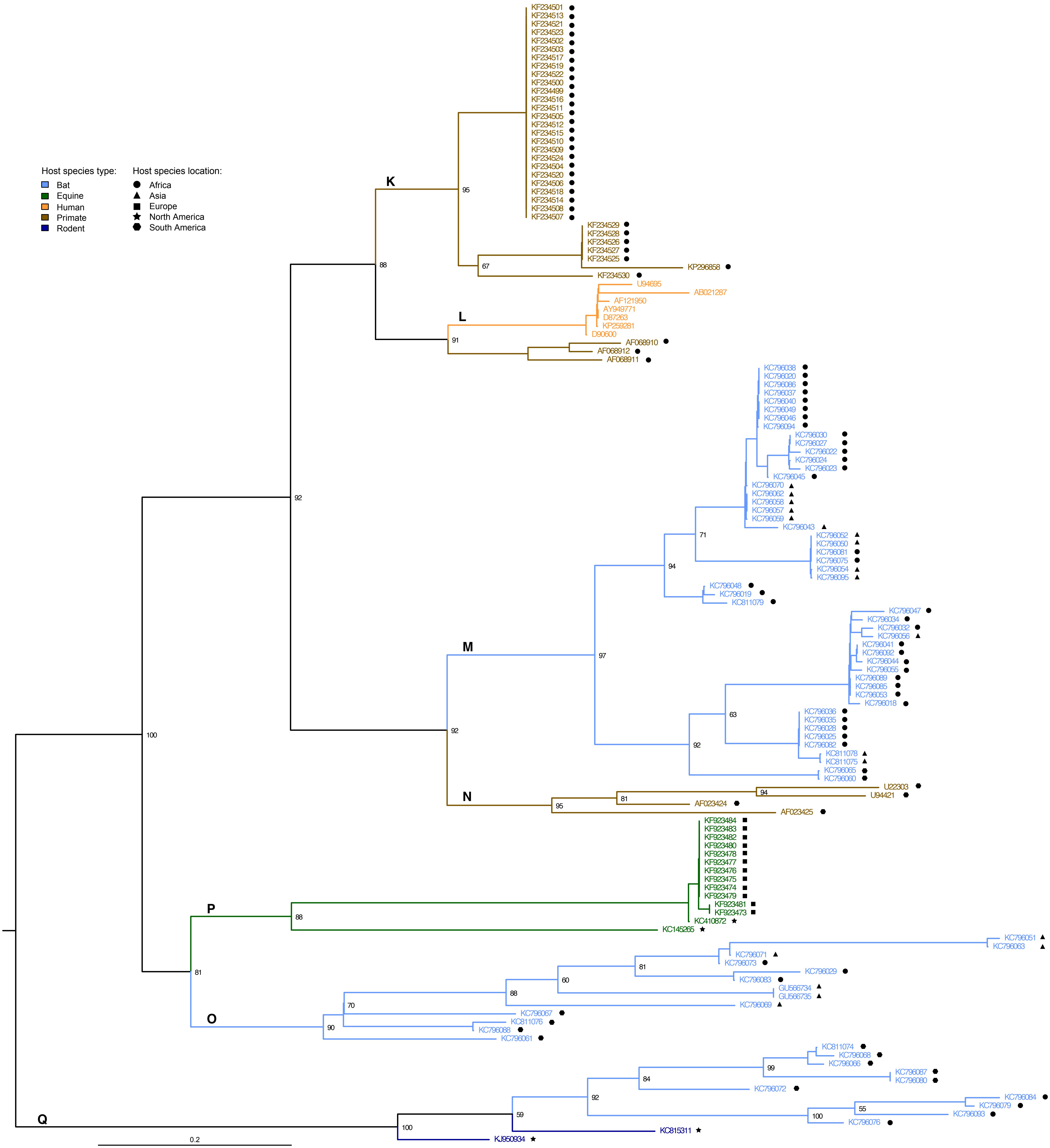

Host species type:
Bat
Equine
Human
Primate
Rodent
Host species location:
Africa
Asia
Europe
North America
South America
K
L
M
N
O
P
Q
KF234501
KF234513
KF234521
KF234523
KF234502
KF234503
KF234517
KF234519
KF234522
KF234500
KF234499
KF234516
KF234511
KF234505
KF234512
KF234515
KF234510
KF234509
KF234524
KF234504
KF234520
KF234506
KF234518
KF234514
KF234508
KF234507
95
KF234529
KF234528
KF234526
KF234527
KF234525
KP296858
67
KF234530
88
U94695
AB021287
AF121950
AY949771
D87263
KP259281
D90600
91
AF068910
AF068912
AF068911
92
KC796038
KC796020
KC796086
KC796037
KC796040
KC796049
KC796046
KC796094
KC796030
KC796027
KC796022
KC796024
KC796023
KC796045
KC796070
KC796062
KC796058
KC796057
KC796059
KC796043
71
KC796052
KC796050
KC796081
KC796075
KC796054
KC796095
94
KC796048
KC796019
KC811079
KC796047
KC796034
KC796032
KC796056
KC796041
KC796092
KC796044
KC796055
KC796089
KC796085
KC796053
KC796018
97
63
KC796036
KC796035
KC796028
KC796025
KC796082
92
KC811078
KC811075
KC796065
KC796060
92
U22303
94
U94421
81
AF023424
95
AF023425
100
KF923484
KF923483
KF923482
KF923480
KF923478
KF923477
KF923476
KF923475
KF923474
KF923479
KF923481
KF923473
88
KC410872
KC145265
KC796051
KC796063
KC796071
KC796073
81
KC796029
60
KC796083
88
GU566734
GU566735
70
KC796069
KC796067
KC811076
90
KC796088
KC796061
81
KC811074
KC796068
KC796066
99
KC796087
KC796080
84
KC796072
92
KC796084
KC796079
55
KC796093
100
KC796076
59
100
KC815311
KJ950934
0.2
