# Supplementary material for: Evolutionary and Phylogenetic Analysis of the Hepaciviruses and Pegiviruses
Source: Genome Biol Evol. 2015 Oct 21;7(11):2996–3008. doi: 10.1093/gbe/evv202 (PMC5635594; doi:10.1093/gbe/evv202)
Supplement: Supplementary Data [file evv202_Supplementary_Data.zip › FigureS1.pdf]

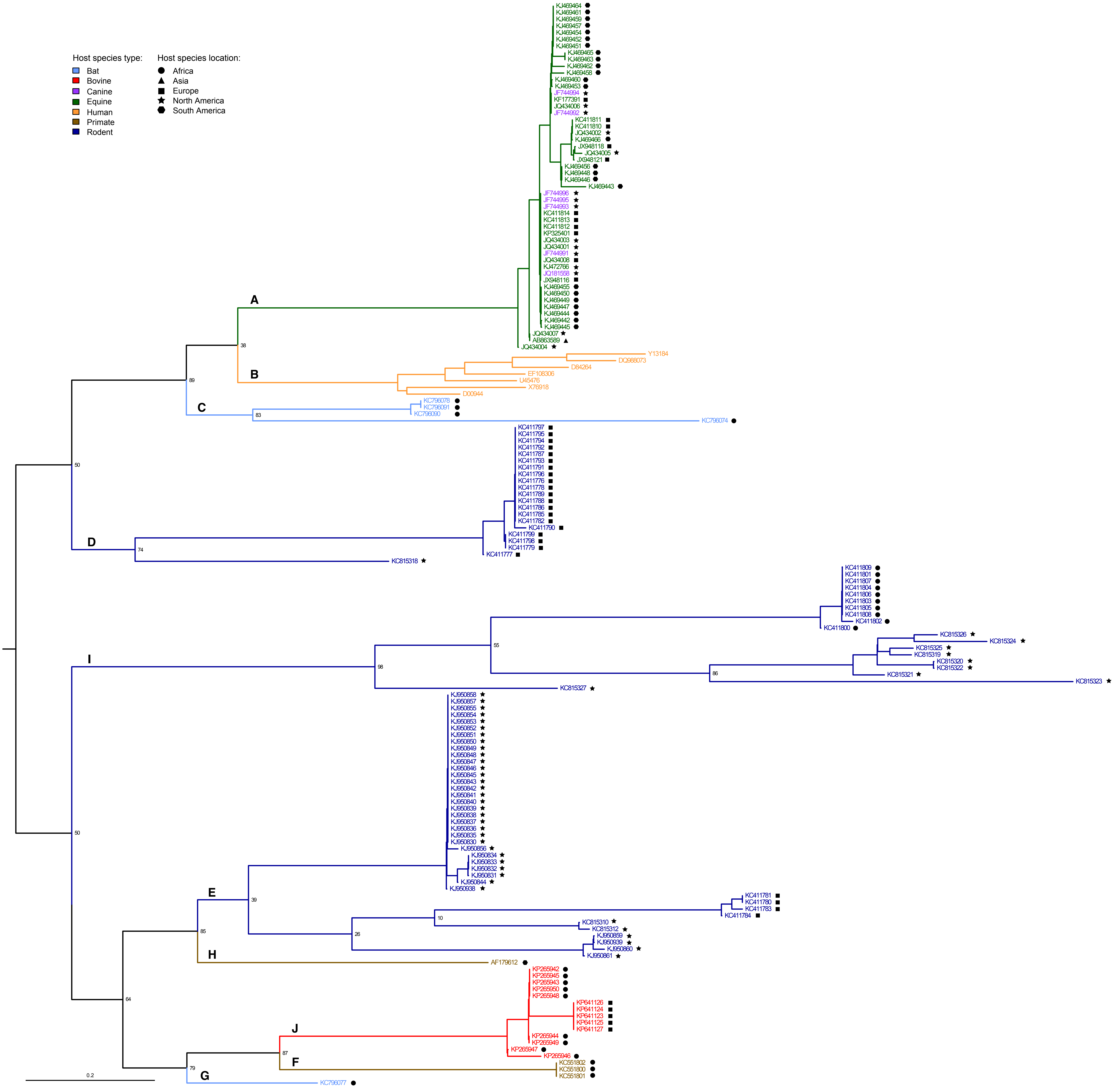

Host species type:
Bat
Bovine
Canine
Equine
Human
Primate
Rodent
Host species location:
Africa
Asia
Europe
North America
South America
A
B
C
D
E
F
G
H
I
J
0.2
KJ469464
KJ469461
KJ469459
KJ469457
KJ469454
KJ469452
KJ469451
KJ469465
KJ469463
KJ469462
KJ469458
KJ469460
KJ469453
JF744994
KF177391
JQ434006
JF744992
KC411811
KC411810
JQ434002
KJ469466
JX948118
JQ434005
JX948121
KJ469456
KJ469448
KJ469446
KJ469443
JF744996
JF744995
JF744993
KC411814
KC411813
KC411812
KP325401
JQ434003
JQ434001
JF744991
JQ434008
KJ472766
JQ181558
JX948116
KJ469455
KJ469450
KJ469449
KJ469447
KJ469444
KJ469442
KJ469445
JQ434007
AB863589
JQ434004
Y13184
DQ988073
D84264
EF108306
U45476
X76918
D00944
KC796078
KC796091
KC796090
KC796074
KC411797
KC411795
KC411794
KC411792
KC411787
KC411793
KC411791
KC411796
KC411776
KC411778
KC411789
KC411788
KC411786
KC411785
KC411782
KC411790
KC411799
KC411798
KC411779
KC411777
KC815318
KC411809
KC411801
KC411807
KC411804
KC411806
KC411803
KC411805
KC411808
KC411802
KC411800
KC815326
KC815324
KC815325
KC815319
KC815320
KC815322
KC815321
KC815323
KC815327
KJ950858
KJ950857
KJ950855
KJ950854
KJ950853
KJ950852
KJ950851
KJ950850
KJ950849
KJ950848
KJ950847
KJ950846
KJ950845
KJ950843
KJ950842
KJ950841
KJ950840
KJ950839
KJ950838
KJ950837
KJ950836
KJ950835
KJ950830
KJ950856
KJ950834
KJ950833
KJ950832
KJ950831
KJ950844
KJ950938
KC411781
KC411780
KC411783
KC411784
KC815310
KC815312
KJ950859
KJ950939
KJ950860
KJ950861
AF179612
KP265942
KP265945
KP265943
KP265950
KP265948
KP641126
KP641124
KP641123
KP641125
KP641127
KP265944
KP265949
KP265947
KP265946
KC551802
KC551800
KC551801
KC796077
38
89
83
50
74
98
55
86
50
39
10
26
85
64
87
79
