# Supplementary material for: Evolutionary and Phylogenetic Analysis of the Hepaciviruses and Pegiviruses
Source: Genome Biol Evol. 2015 Oct 21;7(11):2996–3008. doi: 10.1093/gbe/evv202 (PMC5635594; doi:10.1093/gbe/evv202)
Supplement: Supplementary Data [file evv202_Supplementary_Data.zip › FigureS2.pdf]

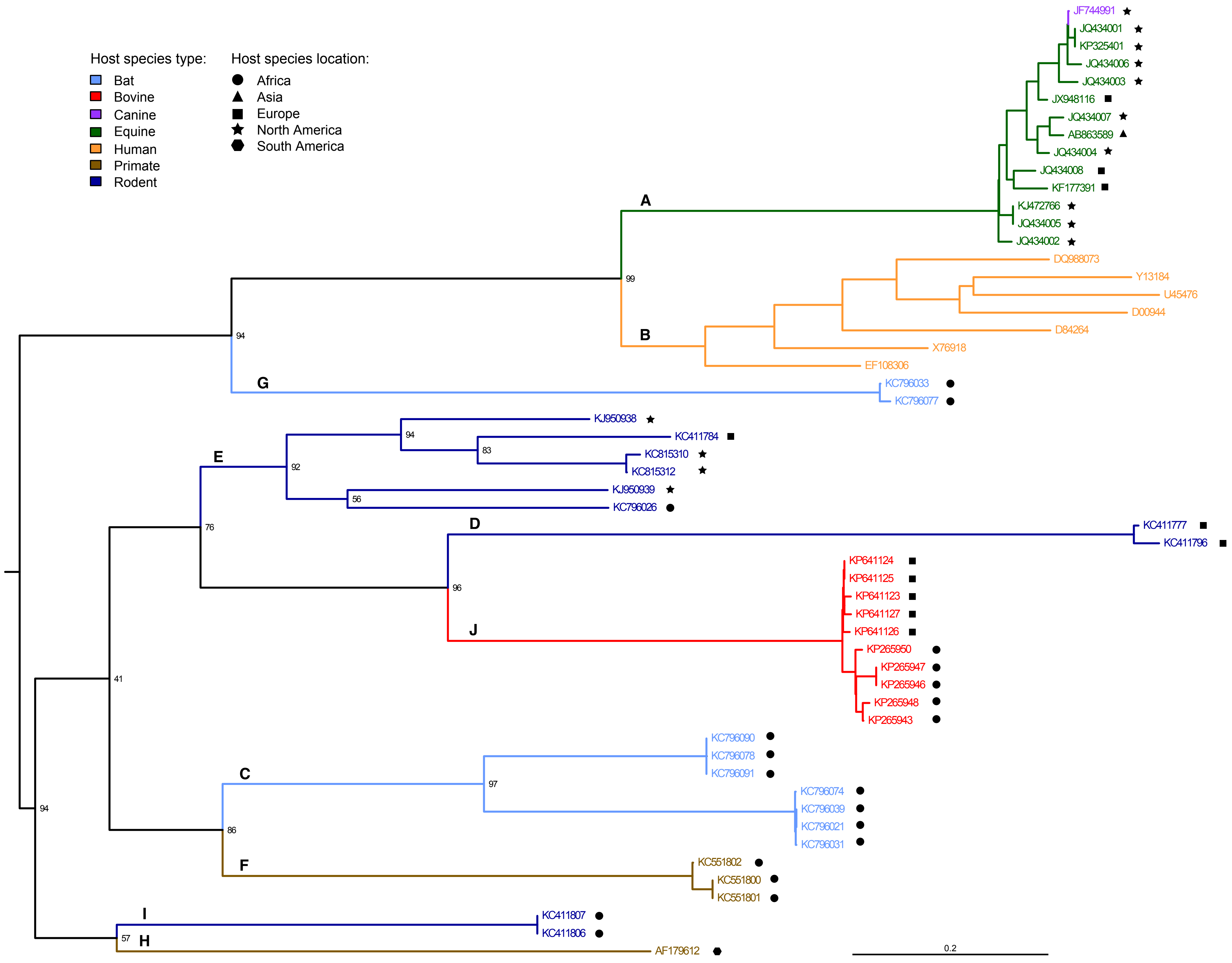
Host species type:
Bat
Bovine
Canine
Equine
Human
Primate
Rodent
Host species location:
Africa
Asia
Europe
North America
South America
A
B
C
D
E
F
G
H
I
J
JF744991
JQ434001
KP325401
JQ434006
JQ434003
JX948116
JQ434007
AB863589
JQ434004
JQ434008
KF177391
KJ472766
JQ434005
JQ434002
DQ988073
Y13184
U45476
D00944
D84264
X76918
EF108306
KC796033
KC796077
KJ950938
KC411784
KC815310
KC815312
KJ950939
KC796026
KC411777
KC411796
KP641124
KP641125
KP641123
KP641127
KP641126
KP265950
KP265947
KP265946
KP265948
KP265943
KC796090
KC796078
KC796091
KC796074
KC796039
KC796021
KC796031
KC551802
KC551800
KC551801
KC411807
KC411806
AF179612
99
94
94
83
92
56
76
96
41
97
86
94
57
0.2
