# Supplementary material for: Evolutionary and Phylogenetic Analysis of the Hepaciviruses and Pegiviruses
Source: Genome Biol Evol. 2015 Oct 21;7(11):2996–3008. doi: 10.1093/gbe/evv202 (PMC5635594; doi:10.1093/gbe/evv202)
Supplement: Supplementary Data [file evv202_Supplementary_Data.zip › FigureS3.pdf]

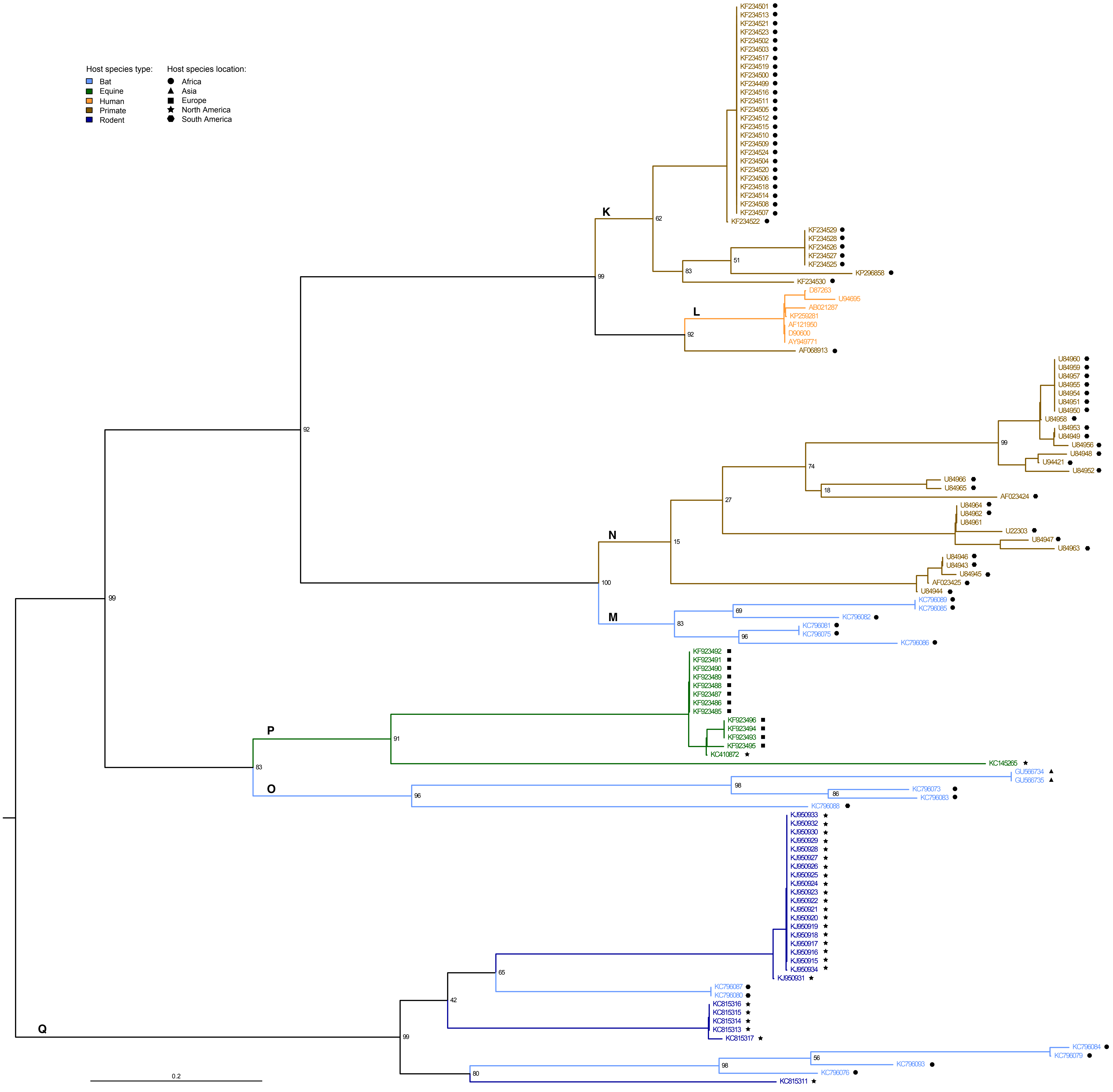

Host species type:
Bat
Equine
Human
Primate
Rodent
Host species location:
Africa
Asia
Europe
North America
South America
K
L
N
M
P
O
Q
0.2
KF234501
KF234513
KF234521
KF234523
KF234502
KF234503
KF234517
KF234519
KF234500
KF234499
KF234516
KF234511
KF234505
KF234512
KF234515
KF234510
KF234509
KF234524
KF234504
KF234520
KF234506
KF234518
KF234514
KF234508
KF234507
KF234522
KF234529
KF234528
KF234526
KF234527
KF234525
KP296858
KF234530
D87263
U94695
AB021287
KP259281
AF121950
D90600
AY949771
AF068913
U84960
U84959
U84957
U84955
U84954
U84951
U84950
U84958
U84953
U84949
U84956
U84948
U94421
U84952
U84966
U84965
AF023424
U84964
U84962
U84961
U22303
U84947
U84963
U84946
U84943
U84945
AF023425
U84944
KC796089
KC796085
KC796082
KC796081
KC796075
KC796086
KF923492
KF923491
KF923490
KF923489
KF923488
KF923487
KF923486
KF923485
KF923496
KF923494
KF923493
KF923495
KC410872
KC145265
GU566734
GU566735
KC796073
KC796083
KC796088
KJ950933
KJ950932
KJ950930
KJ950929
KJ950928
KJ950927
KJ950926
KJ950925
KJ950924
KJ950923
KJ950922
KJ950921
KJ950920
KJ950919
KJ950918
KJ950917
KJ950916
KJ950915
KJ950934
KJ950931
KC796087
KC796080
KC815316
KC815315
KC815314
KC815313
KC815317
KC796084
KC796079
KC796093
KC796076
KC815311
62
51
83
99
92
92
99
74
18
27
15
100
69
83
96
91
83
98
86
96
99
65
42
99
56
98
80
