# Supplementary material for: Evolutionary and Phylogenetic Analysis of the Hepaciviruses and Pegiviruses
Source: Genome Biol Evol. 2015 Oct 21;7(11):2996–3008. doi: 10.1093/gbe/evv202 (PMC5635594; doi:10.1093/gbe/evv202)
Supplement: Supplementary Data [file evv202_Supplementary_Data.zip › Supplementary Figure Legends.doc]

**Supplementary Figure Legends**

**Figure S1.** Phylogeny of the *Hepacivirus* NS3 gene. The tree was obtained from maximum likelihood inference analysis of the amino acid alignment of the NS3 protein. Statistical support for phylogenetic nodes was assessed using a bootstrap approach (100 replicates). Tip and branch labels are coloured by host species type while the sampling location of non-human hosts is denoted by the adjacent symbol. Letters indicate the different *Hepacivirus* lineages discussed in the main text.

**Figure S2.** Phylogeny of the *Hepacivirus* NS5B gene. The tree was obtained from maximum likelihood inference analysis of the amino acid alignment of the NS5B protein. Statistical support for phylogenetic nodes was assessed using a bootstrap approach (100 replicates). Tip and branch labels are coloured by host species type while the sampling location of non-human hosts is denoted by the adjacent symbol. Letters indicate the different *Hepacivirus* lineages discussed in the main text.

**Figure S3.** Phylogeny of the *Pegivirus* NS3 gene. The tree was obtained from maximum likelihood inference analysis of the amino acid alignment of the NS3 protein. Statistical support for phylogenetic nodes was assessed using a bootstrap approach (100 replicates). Tip and branch labels are coloured by host species type while the sampling location of non-human hosts is denoted by the adjacent symbol. Letters indicate the different *Pegivirus* lineages discussed in the main text.

**Figure S4.** Phylogeny of the *Pegivirus* NS5B gene. The tree was obtained from maximum likelihood inference analysis of the amino acid alignment of the NS5B protein. Statistical support for phylogenetic nodes was assessed using a bootstrap approach (100 replicates). Tip and branch labels are coloured by host species type while the sampling location of non-human hosts is denoted by the adjacent symbol. Letters indicate the different *Pegivirus* lineages discussed in the main text.
