# Supplementary material for: Evolutionary and Phylogenetic Analysis of the Hepaciviruses and Pegiviruses
Source: Genome Biol Evol. 2015 Oct 21;7(11):2996–3008. doi: 10.1093/gbe/evv202 (PMC5635594; doi:10.1093/gbe/evv202)
Supplement: Supplementary Data [file evv202_Supplementary_Data.zip › TableS1.doc]

**Table S1.** Sequence data information for hepaciviruses

| **Sequence** | **Accession** | **Isolate** | **Host_species** | **Host_type** | **Locality** | **Region** |
| --- | --- | --- | --- | --- | --- | --- |
| Complete genome | AB863589 | JPN3 | Equus ferus caballus | Equine | Japan | Asia |
|  | AF179612 | GBV-B | Saguinus spp | Primate | Unknown | South America |
|  | D00944 | HCV2 | Homo sapiens | Human | Indonesia | Asia |
|  | D84264 | HCV6 | Homo sapiens | Human | Unknown | Unknown |
|  | DQ988073 | HCV4 | Homo sapiens | Human | Egypt | Africa |
|  | EF108306 | HCV7 | Homo sapiens | Human | Canada | North America |
|  | JF744991 | Unknown | Canis lupus familiaris | Canine | USA | North America |
|  | JQ434001 | NZP-1 | Equus ferus caballus | Equine | USA | North America |
|  | JQ434002 | G1-073 | Equus ferus caballus | Equine | USA | North America |
|  | JQ434003 | A6-006 | Equus ferus caballus | Equine | USA | North America |
|  | JQ434004 | B10-022 | Equus ferus caballus | Equine | USA | North America |
|  | JQ434005 | F8-068 | Equus ferus caballus | Equine | USA | North America |
|  | JQ434006 | G5-077 | Equus ferus caballus | Equine | USA | North America |
|  | JQ434007 | H10-094 | Equus ferus caballus | Equine | USA | North America |
|  | JQ434008 | H3-011 | Equus ferus caballus | Equine | UK | Europe |
|  | JX948116 | EF369_11J | Equus ferus caballus | Equine | UK | Europe |
|  | KC411777 | RMU10-3382 | Myodes glareolus | Rodent | Netherlands | Europe |
|  | KC411784 | NLR07-oct70 | Myodes glareolus | Rodent | Netherlands | Europe |
|  | KC411796 | NLR08-365 | Myodes glareolus | Rodent | Netherlands | Europe |
|  | KC411806 | SAR-3 | Rhabdomys pumilio | Rodent | South Africa | Africa |
|  | KC411807 | SAR-46 | Rhabdomys pumilio | Rodent | South Africa | Africa |
|  | KC551800 | GHV-1_BWC08 | Colobus guereza | Primate | Uganda | Africa |
|  | KC551801 | GHV-1_BWC05 | Colobus guereza | Primate | Uganda | Africa |
|  | KC551802 | GHV-2_BWC04 | Colobus guereza | Primate | Uganda | Africa |
|  | KC796074 | PDB-829 | Hipposideros vittatus | Bat | Kenya | Africa |
|  | KC796077 | PDB-112 | Hipposideros vittatus | Bat | Kenya | Africa |
|  | KC796078 | PDB-491.1 | Otomops martiensseni | Bat | Kenya | Africa |
|  | KC796090 | PDB-452 | Otomops martiensseni | Bat | Kenya | Africa |
|  | KC796091 | PDB-445 | Otomops martiensseni | Bat | Kenya | Africa |
|  | KC815310 | RHV-339 | Peromyscus maniculatus | Rodent | USA | North America |
|  | KC815312 | RHV-089 | Peromyscus maniculatus | Rodent | USA | North America |
|  | KF177391 | DH1 | Equus ferus caballus | Equine | Hungary | Europe |
|  | KJ472766 | WSU-2013 | Equus ferus caballus | Equine | USA | North America |
|  | KJ950938 | NrHV-1_NYC-C12 | Rattus norvegicus | Rodent | USA | North America |
|  | KJ950939 | NrHV-2_NYC-E43 | Rattus norvegicus | Rodent | USA | North America |
|  | KP265943 | GHC25 | Bos taurus | Bovine | Ghana | Africa |
|  | KP265946 | GHC52 | Bos taurus | Bovine | Ghana | Africa |
|  | KP265947 | GHC55 | Bos taurus | Bovine | Ghana | Africa |
|  | KP265948 | GHC85 | Bos taurus | Bovine | Ghana | Africa |
|  | KP265950 | GHC100 | Bos taurus | Bovine | Ghana | Africa |
|  | KP325401 | NZP-1 | Equus ferus caballus | Equine | USA | North America |
|  | KP641123 | B1 | Bos taurus | Bovine | Germany | Europe |
|  | KP641124 | 209 | Bos taurus | Bovine | Germany | Europe |
|  | KP641125 | 379 | Bos taurus | Bovine | Germany | Europe |
|  | KP641126 | 438 | Bos taurus | Bovine | Germany | Europe |
|  | KP641127 | 463 | Bos taurus | Bovine | Germany | Europe |
|  | U45476 | HCV1 | Homo sapiens | Human | Germany | Europe |
|  | X76918 | HCV3 | Homo sapiens | Human | Germany | Europe |
|  | Y13184 | HCV5 | Homo sapiens | Human | South Africa | Africa |
| NS3 | JF744992 | CHV-K217 | Canis lupus familiaris | Canine | USA | North America |
|  | JF744993 | CHV-K188 | Canis lupus familiaris | Canine | USA | North America |
|  | JF744994 | CHV-K180 | Canis lupus familiaris | Canine | USA | North America |
|  | JF744995 | CHV-K154 | Canis lupus familiaris | Canine | USA | North America |
|  | JF744996 | CHV-K136 | Canis lupus familiaris | Canine | USA | North America |
|  | JQ181558 | Unknown | Canis lupus familiaris | Canine | USA | North America |
|  | JX948118 | EF317_98 | Equus ferus caballus | Equine | UK | Europe |
|  | JX948121 | EF330_97 | Equus ferus caballus | Equine | UK | Europe |
|  | KC411776 | RMU10-759 | Myodes glareolus | Rodent | Germany | Europe |
|  | KC411778 | RMU10-3379 | Myodes glareolus | Rodent | Germany | Europe |
|  | KC411779 | RMU10-1602 | Myodes glareolus | Rodent | Germany | Europe |
|  | KC411780 | NLR07-oct88 | Myodes glareolus | Rodent | Netherlands | Europe |
|  | KC411781 | NLR07-oct66 | Myodes glareolus | Rodent | Netherlands | Europe |
|  | KC411782 | NLR07-jun47 | Myodes glareolus | Rodent | Netherlands | Europe |
|  | KC411783 | NLR07-jul47 | Myodes glareolus | Rodent | Netherlands | Europe |
|  | KC411785 | NLR07-jun42 | Myodes glareolus | Rodent | Netherlands | Europe |
|  | KC411786 | NLR07-jun41 | Myodes glareolus | Rodent | Netherlands | Europe |
|  | KC411787 | NLR07-jun40 | Myodes glareolus | Rodent | Netherlands | Europe |
|  | KC411788 | NLR07-jun32 | Myodes glareolus | Rodent | Netherlands | Europe |
|  | KC411789 | NLR07-jun16 | Myodes glareolus | Rodent | Netherlands | Europe |
|  | KC411790 | NLR09-755 | Myodes glareolus | Rodent | Netherlands | Europe |
|  | KC411791 | NLR09-754 | Myodes glareolus | Rodent | Netherlands | Europe |
|  | KC411792 | NLR08-373 | Myodes glareolus | Rodent | Netherlands | Europe |
|  | KC411793 | NLR08-370 | Myodes glareolus | Rodent | Netherlands | Europe |
|  | KC411794 | NLR08-367 | Myodes glareolus | Rodent | Netherlands | Europe |
|  | KC411795 | NLR08-366 | Myodes glareolus | Rodent | Netherlands | Europe |
|  | KC411797 | NLR08-335 | Myodes glareolus | Rodent | Netherlands | Europe |
|  | KC411798 | KS11-259 | Myodes glareolus | Rodent | Germany | Europe |
|  | KC411799 | KS11-211 | Myodes glareolus | Rodent | Germany | Europe |
|  | KC411800 | SAR-A348 | Rhabdomys pumilio | Rodent | South Africa | Africa |
|  | KC411801 | SAR-A23 | Rhabdomys pumilio | Rodent | South Africa | Africa |
|  | KC411802 | SAR-49 | Rhabdomys pumilio | Rodent | South Africa | Africa |
|  | KC411803 | SAR-20 | Rhabdomys pumilio | Rodent | South Africa | Africa |
|  | KC411804 | SAR-5 | Rhabdomys pumilio | Rodent | South Africa | Africa |
|  | KC411805 | SAR-38 | Rhabdomys pumilio | Rodent | South Africa | Africa |
|  | KC411808 | SAR-33 | Rhabdomys pumilio | Rodent | South Africa | Africa |
|  | KC411809 | SAR-A25 | Rhabdomys pumilio | Rodent | South Africa | Africa |
|  | KC411810 | GER-Eq179 | Equus ferus caballus | Equine | Germany | Europe |
|  | KC411811 | GER-Eq285 | Equus ferus caballus | Equine | Germany | Europe |
|  | KC411812 | GER-Eq143 | Equus ferus caballus | Equine | Germany | Europe |
|  | KC411813 | GER-Eq120 | Equus ferus caballus | Equine | Germany | Europe |
|  | KC411814 | GER-Eq105 | Equus ferus caballus | Equine | Germany | Europe |
|  | KC815318 | RHV-ch2110 | Chaetodipus hispidus | Rodent | USA | North America |
|  | KC815319 | RHV-pm5263 | Peromyscus maniculatus | Rodent | USA | North America |
|  | KC815320 | RHV-pm3243 | Peromyscus maniculatus | Rodent | USA | North America |
|  | KC815321 | RHV-pm5198 | Peromyscus maniculatus | Rodent | USA | North America |
|  | KC815322 | RHV-pm3252 | Peromyscus maniculatus | Rodent | USA | North America |
|  | KC815323 | RHV-nl4106 | Neotoma lepida | Rodent | USA | North America |
|  | KC815324 | RHV-pm4062 | Peromyscus maniculatus | Rodent | USA | North America |
|  | KC815325 | RHV-pm4109 | Peromyscus maniculatus | Rodent | USA | North America |
|  | KC815326 | RHV-pm4144 | Peromyscus maniculatus | Rodent | USA | North America |
|  | KC815327 | RHV-pm3038 | Peromyscus maniculatus | Rodent | USA | North America |
|  | KJ469442 | AS8912 | Equus ferus caballus | Equine | Brazil | South America |
|  | KJ469443 | AS8927 | Equus ferus caballus | Equine | Brazil | South America |
|  | KJ469444 | AS8930 | Equus ferus caballus | Equine | Brazil | South America |
|  | KJ469445 | AS8932 | Equus ferus caballus | Equine | Brazil | South America |
|  | KJ469446 | AS8938 | Equus ferus caballus | Equine | Brazil | South America |
|  | KJ469447 | AS8939 | Equus ferus caballus | Equine | Brazil | South America |
|  | KJ469448 | AS8940 | Equus ferus caballus | Equine | Brazil | South America |
|  | KJ469449 | AS8941 | Equus ferus caballus | Equine | Brazil | South America |
|  | KJ469450 | AS8943 | Equus ferus caballus | Equine | Brazil | South America |
|  | KJ469451 | AS8990 | Equus ferus caballus | Equine | Brazil | South America |
|  | KJ469452 | AS9008 | Equus ferus caballus | Equine | Brazil | South America |
|  | KJ469453 | AS9016 | Equus ferus caballus | Equine | Brazil | South America |
|  | KJ469454 | AS9019 | Equus ferus caballus | Equine | Brazil | South America |
|  | KJ469455 | AS9068 | Equus ferus caballus | Equine | Brazil | South America |
|  | KJ469456 | AS9069 | Equus ferus caballus | Equine | Brazil | South America |
|  | KJ469457 | AS9186 | Equus ferus caballus | Equine | Brazil | South America |
|  | KJ469458 | AS9201 | Equus ferus caballus | Equine | Brazil | South America |
|  | KJ469459 | AS9218 | Equus ferus caballus | Equine | Brazil | South America |
|  | KJ469460 | AS9264 | Equus ferus caballus | Equine | Brazil | South America |
|  | KJ469461 | AS9293 | Equus ferus caballus | Equine | Brazil | South America |
|  | KJ469462 | AS9298 | Equus ferus caballus | Equine | Brazil | South America |
|  | KJ469463 | AS9308 | Equus ferus caballus | Equine | Brazil | South America |
|  | KJ469464 | AS9322 | Equus ferus caballus | Equine | Brazil | South America |
|  | KJ469465 | AS9328 | Equus ferus caballus | Equine | Brazil | South America |
|  | KJ469466 | AS9330 | Equus ferus caballus | Equine | Brazil | South America |
|  | KJ950830 | NrHV-1_NYC-C12 | Rattus norvegicus | Rodent | USA | North America |
|  | KJ950831 | NrHV-1_NYC-A16 | Rattus norvegicus | Rodent | USA | North America |
|  | KJ950832 | NrHV-1_NYC-A17 | Rattus norvegicus | Rodent | USA | North America |
|  | KJ950833 | NrHV-1_NYC-A22 | Rattus norvegicus | Rodent | USA | North America |
|  | KJ950834 | NrHV-1_NYC-A8 | Rattus norvegicus | Rodent | USA | North America |
|  | KJ950835 | NrHV-1_NYC-C10 | Rattus norvegicus | Rodent | USA | North America |
|  | KJ950836 | NrHV-1_NYC-C11a | Rattus norvegicus | Rodent | USA | North America |
|  | KJ950837 | NrHV-1_NYC-C15 | Rattus norvegicus | Rodent | USA | North America |
|  | KJ950838 | NrHV-1_NYC-C2 | Rattus norvegicus | Rodent | USA | North America |
|  | KJ950839 | NrHV-1_NYC-C4 | Rattus norvegicus | Rodent | USA | North America |
|  | KJ950840 | NrHV-1_NYC-C5 | Rattus norvegicus | Rodent | USA | North America |
|  | KJ950841 | NrHV-1_NYC-C6 | Rattus norvegicus | Rodent | USA | North America |
|  | KJ950842 | NrHV-1_NYC-C8 | Rattus norvegicus | Rodent | USA | North America |
|  | KJ950843 | NrHV-1_NYC-C9 | Rattus norvegicus | Rodent | USA | North America |
|  | KJ950844 | NrHV-1_NYC-D1 | Rattus norvegicus | Rodent | USA | North America |
|  | KJ950845 | NrHV-1_NYC-D15 | Rattus norvegicus | Rodent | USA | North America |
|  | KJ950846 | NrHV-1_NYC-D17 | Rattus norvegicus | Rodent | USA | North America |
|  | KJ950847 | NrHV-1_NYC-D2 | Rattus norvegicus | Rodent | USA | North America |
|  | KJ950848 | NrHV-1_NYC-D23 | Rattus norvegicus | Rodent | USA | North America |
|  | KJ950849 | NrHV-1_NYC-D4 | Rattus norvegicus | Rodent | USA | North America |
|  | KJ950850 | NrHV-1_NYC-D9 | Rattus norvegicus | Rodent | USA | North America |
|  | KJ950851 | NrHV-1_NYC-E13 | Rattus norvegicus | Rodent | USA | North America |
|  | KJ950852 | NrHV-1_NYC-E31 | Rattus norvegicus | Rodent | USA | North America |
|  | KJ950853 | NrHV-1_NYC-E35a | Rattus norvegicus | Rodent | USA | North America |
|  | KJ950854 | NrHV-1_NYC-E43 | Rattus norvegicus | Rodent | USA | North America |
|  | KJ950855 | NrHV-1_NYC-E46 | Rattus norvegicus | Rodent | USA | North America |
|  | KJ950856 | NrHV-1_NYC-E7 | Rattus norvegicus | Rodent | USA | North America |
|  | KJ950857 | NrHV-1_NYC-E8 | Rattus norvegicus | Rodent | USA | North America |
|  | KJ950858 | NrHV-1_NYC-E9 | Rattus norvegicus | Rodent | USA | North America |
|  | KJ950859 | NrHV-2_NYC-E27 | Rattus norvegicus | Rodent | USA | North America |
|  | KJ950860 | NrHV-2_NYC-E46 | Rattus norvegicus | Rodent | USA | North America |
|  | KJ950861 | NrHV-2_NYC-E7 | Rattus norvegicus | Rodent | USA | North America |
|  | KP265942 | GHC11 | Bos taurus | Bovine | Ghana | Africa |
|  | KP265944 | GHC32 | Bos taurus | Bovine | Ghana | Africa |
|  | KP265945 | GHC37 | Bos taurus | Bovine | Ghana | Africa |
|  | KP265949 | GHC87 | Bos taurus | Bovine | Ghana | Africa |
| NS5B | KC796021 | PDB-632B | Hipposideros vittatus | Bat | Kenya | Africa |
|  | KC796026 | PDB-638B | Scotoecus spp | Bat | Kenya | Africa |
|  | KC796031 | PDB-261 | Hipposideros vittatus | Bat | Kenya | Africa |
|  | KC796033 | PDB-113 | Hipposideros vittatus | Bat | Kenya | Africa |
|  | KC796039 | PDB-830 | Hipposideros vittatus | Bat | Kenya | Africa |
