# Supplementary material for: Evolutionary and Phylogenetic Analysis of the Hepaciviruses and Pegiviruses
Source: Genome Biol Evol. 2015 Oct 21;7(11):2996–3008. doi: 10.1093/gbe/evv202 (PMC5635594; doi:10.1093/gbe/evv202)
Supplement: Supplementary Data [file evv202_Supplementary_Data.zip › TableS2.doc]

**Table S2.** Sequence data information for pegiviruses

| **Sequence** | **Accession** | **Isolate** | **Host species** | **Host type** | **Locality** | **Region** |
| --- | --- | --- | --- | --- | --- | --- |
| Complete genome | AB021287 | GBV-C4 | Homo sapiens | Human | Myanmar or Vietnam | Asia |
|  | AF023424 | GBV-A_mx70047 | Callithrix hybrid | Primate | Unknown | South America |
|  | AF023425 | GBV-A_tri1122 | Aotus trivirgatus | Primate | Unknown | South America |
|  | AF121950 | GBV-C6 | Homo sapiens | Human | USA | North America |
|  | AY949771 | GBV-C5 | Homo sapiens | Human | South Africa | Africa |
|  | D87263 | GBV-C3 | Homo sapiens | Human | Unknown | Unknown |
|  | D90600 | GBV-C2 | Homo sapiens | Human | Japan | Asia |
|  | GU566734 | GBV-D_68 | Pteropus giganteus | Bat | Bangladesh | Asia |
|  | GU566735 | GBV-D_93 | Pteropus giganteus | Bat | Bangladesh | Asia |
|  | KC145265 | HorseA1_serum | Equus ferus caballus | Equine | USA | North America |
|  | KC410872 | C0035 | Equus ferus caballus | Equine | USA | North America |
|  | KC796073 | PDB-303 | Megaloglossus woermanni | Bat | Democratic Republic of Congo | Africa |
|  | KC796075 | PDB-106 | Eidolon helvum | Bat | Cameroon | Africa |
|  | KC796076 | PDB-620 | Scotophilus dinganii | Bat | Kenya | Africa |
|  | KC796079 | PDB-99 | Taphozous spp | Bat | Cameroon | Africa |
|  | KC796080 | PDB-1698 | Carollia perspicillata | Bat | Guatemala | South America |
|  | KC796081 | PDB-737B | Eidolon helvum | Bat | Kenya | Africa |
|  | KC796082 | PDB-24 | Chaerephon spp | Bat | Cameroon | Africa |
|  | KC796083 | PDB-694 | Eidolon helvum | Bat | Nigeria | Africa |
|  | KC796084 | PDB-76.1 | Taphozous spp | Bat | Cameroon | Africa |
|  | KC796085 | PDB-534 | Otomops martiensseni | Bat | Kenya | Africa |
|  | KC796086 | PDB-838 | Rousettus aegyptiacus | Bat | Kenya | Africa |
|  | KC796087 | PDB-1734 | Carollia perspicillata | Bat | Guatemala | South America |
|  | KC796088 | PDB-1715 | Sturnira lilium | Bat | Guatemala | South America |
|  | KC796089 | PDB-491.2 | Otomops martiensseni | Bat | Kenya | Africa |
|  | KC796093 | PDB-34.1 | Mops condylurus | Bat | Democratic Republic of Congo | Africa |
|  | KC815311 | CC61 | Neotoma lepida | Rodent | USA | North America |
|  | KF234499 | SPgVkrc_RC06 | Piliocolobus tephrosceles | Primate | Uganda | Africa |
|  | KF234500 | SPgVkrc_RC33 | Piliocolobus tephrosceles | Primate | Uganda | Africa |
|  | KF234501 | SPgVkrc_RC08 | Piliocolobus tephrosceles | Primate | Uganda | Africa |
|  | KF234502 | SPgVkrc_RC29 | Piliocolobus tephrosceles | Primate | Uganda | Africa |
|  | KF234503 | SPgVkrc_RC32 | Piliocolobus tephrosceles | Primate | Uganda | Africa |
|  | KF234504 | SPgVkrc_RC40 | Piliocolobus tephrosceles | Primate | Uganda | Africa |
|  | KF234505 | SPgVkrc_RC01 | Piliocolobus tephrosceles | Primate | Uganda | Africa |
|  | KF234506 | SPgVkrc_RC58 | Piliocolobus tephrosceles | Primate | Uganda | Africa |
|  | KF234507 | SPgVkrc_RC31 | Piliocolobus tephrosceles | Primate | Uganda | Africa |
|  | KF234508 | SPgVkrc_RC41 | Piliocolobus tephrosceles | Primate | Uganda | Africa |
|  | KF234509 | SPgVkrc_RC34 | Piliocolobus tephrosceles | Primate | Uganda | Africa |
|  | KF234510 | SPgVkrc_RC07 | Piliocolobus tephrosceles | Primate | Uganda | Africa |
|  | KF234511 | SPgVkrc_RC52 | Piliocolobus tephrosceles | Primate | Uganda | Africa |
|  | KF234512 | SPgVkrc_RC53 | Piliocolobus tephrosceles | Primate | Uganda | Africa |
|  | KF234513 | SPgVkrc_RC14 | Piliocolobus tephrosceles | Primate | Uganda | Africa |
|  | KF234514 | SPgVkrc_RC61 | Piliocolobus tephrosceles | Primate | Uganda | Africa |
|  | KF234515 | SPgVkrc_RC09 | Piliocolobus tephrosceles | Primate | Uganda | Africa |
|  | KF234516 | SPgVkrc_RC51 | Piliocolobus tephrosceles | Primate | Uganda | Africa |
|  | KF234517 | SPgVkrc_RC47 | Piliocolobus tephrosceles | Primate | Uganda | Africa |
|  | KF234518 | SPgVkrc_RC60 | Piliocolobus tephrosceles | Primate | Uganda | Africa |
|  | KF234519 | SPgVkrc_RC19 | Piliocolobus tephrosceles | Primate | Uganda | Africa |
|  | KF234520 | SPgVkrc_RC54 | Piliocolobus tephrosceles | Primate | Uganda | Africa |
|  | KF234521 | SPgVkrc_RC43 | Piliocolobus tephrosceles | Primate | Uganda | Africa |
|  | KF234522 | SPgVkrc_RC26 | Piliocolobus tephrosceles | Primate | Uganda | Africa |
|  | KF234523 | SPgVkrc_RC13 | Piliocolobus tephrosceles | Primate | Uganda | Africa |
|  | KF234524 | SPgVkrc_RC59 | Piliocolobus tephrosceles | Primate | Uganda | Africa |
|  | KF234525 | SPgVkrtg_RT03 | Cercopithecus ascanius | Primate | Uganda | Africa |
|  | KF234526 | SPgVkrtg_RT06 | Cercopithecus ascanius | Primate | Uganda | Africa |
|  | KF234527 | SPgVkrtg_RT08 | Cercopithecus ascanius | Primate | Uganda | Africa |
|  | KF234528 | SPgVkrtg_RT05 | Cercopithecus ascanius | Primate | Uganda | Africa |
|  | KF234529 | SPgVkrtg_RT11 | Cercopithecus ascanius | Primate | Uganda | Africa |
|  | KF234530 | SPgVkob_OB23 | Papio anubis | Primate | Uganda | Africa |
|  | KJ950934 | NYC-E13 | Rattus norvegicus | Rodent | USA | North America |
|  | KP259281 | HPgV2_H40048 | Homo sapiens | Human | Denmark | Europe |
|  | KP296858 | VGA00020 | Chlorocebus sabaeus | Primate | Unknown | Africa |
|  | U22303 | GBV-A | Saguinus spp | Primate | Unknown | South America |
|  | U94421 | GBV-A_Alab | Saguinus labiatus | Primate | Unknown | South America |
|  | U94695 | GBV-C1 | Homo sapiens | Human | China | Asia |
| NS3 | AF068913 | GBV-C-like_33 | Pan troglodytes | Primate | Unknown | Africa |
|  | KC815313 | pm6087 | Peromyscus maniculatus | Rodent | USA | North America |
|  | KC815314 | pm6197 | Peromyscus maniculatus | Rodent | USA | North America |
|  | KC815315 | pm6073 | Peromyscus maniculatus | Rodent | USA | North America |
|  | KC815316 | pm6041 | Peromyscus maniculatus | Rodent | USA | North America |
|  | KC815317 | pm5226 | Peromyscus maniculatus | Rodent | USA | North America |
|  | KF923485 | EF607_12 | Equus ferus caballus | Equine | UK | Europe |
|  | KF923486 | EF604_12 | Equus ferus caballus | Equine | UK | Europe |
|  | KF923487 | EF610_12 | Equus ferus caballus | Equine | UK | Europe |
|  | KF923488 | EF381_97 | Equus ferus caballus | Equine | UK | Europe |
|  | KF923489 | EF382_97 | Equus ferus caballus | Equine | UK | Europe |
|  | KF923490 | EF383_97 | Equus ferus caballus | Equine | UK | Europe |
|  | KF923491 | EF384_97 | Equus ferus caballus | Equine | UK | Europe |
|  | KF923492 | EF385_97 | Equus ferus caballus | Equine | UK | Europe |
|  | KF923493 | EF_FR_15 | Equus ferus caballus | Equine | France | Europe |
|  | KF923494 | EF_FR_13 | Equus ferus caballus | Equine | France | Europe |
|  | KF923495 | EF_FR_7 | Equus ferus caballus | Equine | France | Europe |
|  | KF923496 | EF_FR_11 | Equus ferus caballus | Equine | France | Europe |
|  | KJ950915 | NYC-C11 | Rattus norvegicus | Rodent | USA | North America |
|  | KJ950916 | NYC-C19 | Rattus norvegicus | Rodent | USA | North America |
|  | KJ950917 | NYC-C3 | Rattus norvegicus | Rodent | USA | North America |
|  | KJ950918 | NYC-C6 | Rattus norvegicus | Rodent | USA | North America |
|  | KJ950919 | NYC-C8 | Rattus norvegicus | Rodent | USA | North America |
|  | KJ950920 | NYC-C9 | Rattus norvegicus | Rodent | USA | North America |
|  | KJ950921 | NYC-D1 | Rattus norvegicus | Rodent | USA | North America |
|  | KJ950922 | NYC-D15 | Rattus norvegicus | Rodent | USA | North America |
|  | KJ950923 | NYC-D17 | Rattus norvegicus | Rodent | USA | North America |
|  | KJ950924 | NYC-D2 | Rattus norvegicus | Rodent | USA | North America |
|  | KJ950925 | NYC-D23 | Rattus norvegicus | Rodent | USA | North America |
|  | KJ950926 | NYC-D3 | Rattus norvegicus | Rodent | USA | North America |
|  | KJ950927 | NYC-E23 | Rattus norvegicus | Rodent | USA | North America |
|  | KJ950928 | NYC-E26 | Rattus norvegicus | Rodent | USA | North America |
|  | KJ950929 | NYC-E27 | Rattus norvegicus | Rodent | USA | North America |
|  | KJ950930 | NYC-E31 | Rattus norvegicus | Rodent | USA | North America |
|  | KJ950931 | NYC-E35 | Rattus norvegicus | Rodent | USA | North America |
|  | KJ950932 | NYC-E43 | Rattus norvegicus | Rodent | USA | North America |
|  | KJ950933 | NYC-E7 | Rattus norvegicus | Rodent | USA | North America |
|  | U84943 | GBV-A-like_27 | Aotus trivirgatus | Primate | Unknown | South America |
|  | U84944 | GBV-A-like_924 | Aotus trivirgatus | Primate | Unknown | South America |
|  | U84945 | GBV-A-like_925 | Aotus trivirgatus | Primate | Unknown | South America |
|  | U84946 | GBV-A-like_933 | Aotus trivirgatus | Primate | Unknown | South America |
|  | U84947 | GBV-A-like_119 | Saguinus labiatus | Primate | Unknown | South America |
|  | U84948 | GBV-A-like_140 | Saguinus labiatus | Primate | Unknown | South America |
|  | U84949 | GBV-A-like_156 | Saguinus mystax | Primate | Unknown | South America |
|  | U84950 | GBV-A-like_737 | Saguinus mystax | Primate | Unknown | South America |
|  | U84951 | GBV-A-like_749 | Saguinus mystax | Primate | Unknown | South America |
|  | U84952 | GBV-A-like_750 | Saguinus mystax | Primate | Unknown | South America |
|  | U84953 | GBV-A-like_760 | Saguinus mystax | Primate | Unknown | South America |
|  | U84954 | GBV-A-like_768 | Saguinus mystax | Primate | Unknown | South America |
|  | U84955 | GBV-A-like_782 | Saguinus mystax | Primate | Unknown | South America |
|  | U84956 | GBV-A-like_795 | Saguinus mystax | Primate | Unknown | South America |
|  | U84957 | GBV-A-like_799 | Saguinus mystax | Primate | Unknown | South America |
|  | U84958 | GBV-A-like_805 | Saguinus mystax | Primate | Unknown | South America |
|  | U84959 | GBV-A-like_810 | Saguinus mystax | Primate | Unknown | South America |
|  | U84960 | GBV-A-like_823 | Saguinus mystax | Primate | Unknown | South America |
|  | U84961 | GBV-A-like_17 | Saguinus nigricollis | Primate | Unknown | South America |
|  | U84962 | GBV-A-like_18 | Saguinus nigricollis | Primate | Unknown | South America |
|  | U84963 | GBV-A-like_95 | Saguinus nigricollis | Primate | Unknown | South America |
|  | U84964 | GBV-A-like_98 | Saguinus nigricollis | Primate | Unknown | South America |
|  | U84965 | GBV-A-like_100 | Saguinus oedipus | Primate | Unknown | South America |
|  | U84966 | GBV-A-like_14 | Saguinus oedipus | Primate | Unknown | South America |
| NS5B | AF068910 | GBV-C-like_23 | Pan troglodytes | Primate | Unknown | Africa |
|  | AF068911 | GBV-C-like_30 | Pan troglodytes | Primate | Unknown | Africa |
|  | AF068912 | GBV-C-like_33 | Pan troglodytes | Primate | Unknown | Africa |
|  | KC796018 | PDB-664 | Eidolon helvum | Bat | Nigeria | Africa |
|  | KC796019 | PDB-702 | Rousettus aegyptiacus | Bat | Nigeria | Africa |
|  | KC796020 | PDB-623 | Unknown | Bat | Nigeria | Africa |
|  | KC796022 | PDB-706 | Rousettus aegyptiacus | Bat | Nigeria | Africa |
|  | KC796023 | PDB-692 | Eidolon helvum | Bat | Nigeria | Africa |
|  | KC796024 | PDB-34.2 | Mops condylurus | Bat | Democratic Republic of Congo | Africa |
|  | KC796025 | PDB-28A | Chaerephon spp | Bat | Cameroon | Africa |
|  | KC796027 | PDB-423 | Chaerephon spp | Bat | Kenya | Africa |
|  | KC796028 | PDB-47 | Mops condylurus | Bat | Democratic Republic of Congo | Africa |
|  | KC796029 | PDB-366 | Eidolon helvum | Bat | Democratic Republic of Congo | Africa |
|  | KC796030 | PDB-76.2 | Taphozous spp | Bat | Cameroon | Africa |
|  | KC796032 | PDB-401 | Epomophorus labiatus | Bat | Kenya | Africa |
|  | KC796034 | PDB-690 | Eidolon helvum | Bat | Nigeria | Africa |
|  | KC796035 | PDB-130 | Hipposideros vittatus | Bat | Kenya | Africa |
|  | KC796036 | PDB-41A | Chaerephon spp | Bat | Cameroon | Africa |
|  | KC796037 | PDB-883A | Rousettus aegyptiacus | Bat | Nigeria | Africa |
|  | KC796038 | PDB-895 | Rousettus aegyptiacus | Bat | Nigeria | Africa |
|  | KC796040 | PDB-798 | Unknown | Bat | Nigeria | Africa |
|  | KC796041 | PDB-725 | Eidolon helvum | Bat | Nigeria | Africa |
|  | KC796043 | PRB-1006.2 | Pteropus giganteus | Bat | Bangladesh | Asia |
|  | KC796044 | PDB-722 | Rousettus aegyptiacus | Bat | Nigeria | Africa |
|  | KC796045 | PDB-840 | Rousettus aegyptiacus | Bat | Kenya | Africa |
|  | KC796046 | PDB-854 | Rousettus aegyptiacus | Bat | Kenya | Africa |
|  | KC796047 | PDB-935 | Unknown | Bat | Nigeria | Africa |
|  | KC796048 | PDB-903 | Hipposideros gigas | Bat | Nigeria | Africa |
|  | KC796049 | PDB-898 | Rousettus aegyptiacus | Bat | Nigeria | Africa |
|  | KC796050 | PRB-940 | Pteropus giganteus | Bat | Bangladesh | Asia |
|  | KC796051 | PRB-891 | Pteropus giganteus | Bat | Bangladesh | Asia |
|  | KC796052 | PRB-1006.1 | Pteropus giganteus | Bat | Bangladesh | Asia |
|  | KC796053 | PDB-716 | Eidolon helvum | Bat | Nigeria | Africa |
|  | KC796054 | PRB-1085 | Pteropus giganteus | Bat | Bangladesh | Asia |
|  | KC796055 | PDB-743 | Rousettus aegyptiacus | Bat | Nigeria | Africa |
|  | KC796056 | PRB-1129 | Pteropus giganteus | Bat | Bangladesh | Asia |
|  | KC796057 | PRB-1515 | Pteropus giganteus | Bat | Bangladesh | Asia |
|  | KC796058 | PRB-1549 | Pteropus giganteus | Bat | Bangladesh | Asia |
|  | KC796059 | PRB-1520 | Pteropus giganteus | Bat | Bangladesh | Asia |
|  | KC796060 | PMX-1376-re | Nyctinomops macrotis | Bat | Mexico | South America |
|  | KC796061 | PDB-1699 | Desmodus rotundus | Bat | Guatemala | South America |
|  | KC796062 | PRB-1522 | Pteropus giganteus | Bat | Bangladesh | Asia |
|  | KC796063 | PRB-1554 | Pteropus giganteus | Bat | Bangladesh | Asia |
|  | KC796065 | PMX-1376-pl | Nyctinomops macrotis | Bat | Mexico | South America |
|  | KC796066 | PMX-1641 | Glossophaga comissarisi | Bat | Mexico | South America |
|  | KC796067 | PMX-1588 | Carollia perspicillata | Bat | Mexico | South America |
|  | KC796068 | PMX-1508 | Trachops cirrhosus | Bat | Mexico | South America |
|  | KC796069 | PRB-1447 | Pteropus giganteus | Bat | Bangladesh | Asia |
|  | KC796070 | PRB-1547 | Pteropus giganteus | Bat | Bangladesh | Asia |
|  | KC796071 | PRB-1479 | Pteropus giganteus | Bat | Bangladesh | Asia |
|  | KC796072 | PMX-1615 | Artibeus watsoni | Bat | Mexico | South America |
|  | KC796092 | PDB-152 | Hipposideros vittatus | Bat | Kenya | Africa |
|  | KC796094 | PDB-921 | Rousettus aegyptiacus | Bat | Nigeria | Africa |
|  | KC796095 | PRB-1170 | Pteropus giganteus | Bat | Bangladesh | Asia |
|  | KC811074 | PMX-1503 | Trachops cirrhosus | Bat | Mexico | South America |
|  | KC811075 | PRB-978 | Pteropus giganteus | Bat | Bangladesh | Asia |
|  | KC811076 | PMX-1608 | Sturnira ludovici | Bat | Mexico | South America |
|  | KC811078 | PRB1476 | Pteropus giganteus | Bat | Bangladesh | Asia |
|  | KC811079 | PDB-307.lu | Rousettus aegyptiacus | Bat | Nigeria | Africa |
|  | KF923473 | EF385_97 | Equus ferus caballus | Equine | UK | Europe |
|  | KF923474 | EF382_97 | Equus ferus caballus | Equine | UK | Europe |
|  | KF923475 | EF381_97 | Equus ferus caballus | Equine | UK | Europe |
|  | KF923476 | EF610_12 | Equus ferus caballus | Equine | UK | Europe |
|  | KF923477 | EF383_97 | Equus ferus caballus | Equine | UK | Europe |
|  | KF923478 | EF384_97 | Equus ferus caballus | Equine | UK | Europe |
|  | KF923479 | EF607_12 | Equus ferus caballus | Equine | UK | Europe |
|  | KF923480 | EF604_12 | Equus ferus caballus | Equine | UK | Europe |
|  | KF923481 | EF_FR_7 | Equus ferus caballus | Equine | France | Europe |
|  | KF923482 | EF_FR_11 | Equus ferus caballus | Equine | France | Europe |
|  | KF923483 | EF_FR_15 | Equus ferus caballus | Equine | France | Europe |
|  | KF923484 | EF_FR_13 | Equus ferus caballus | Equine | France | Europe |
